# IMP1 KH1 and KH2 domains create a structural platform with unique RNA recognition and re-modelling properties

**DOI:** 10.1093/nar/gkz136

**Published:** 2019-03-13

**Authors:** Robert Dagil, Neil J Ball, Roksana W Ogrodowicz, Fruzsina Hobor, Andrew G Purkiss, Geoff Kelly, Stephen R Martin, Ian A Taylor, Andres Ramos

**Affiliations:** 1 Research Department of Structural and Molecular Biology, University College London, Darwin Building, Gower Street, London WC1E 6XA, UK; 2 Macromolecular Structure Laboratory, The Francis Crick Institute, 1 Midland Road, London NW1 1AT, UK; 3 Structural Biology Science Technology Platform, The Francis Crick Institute, 1 Midland Road, London NW1 1AT, UK; 4 MRC Biomedical NMR Centre, The Francis Crick Institute, 1 Midland Road, London NW1 1AT, UK

## Abstract

IGF2 mRNA-binding protein 1 (IMP1) is a key regulator of messenger RNA (mRNA) metabolism and transport in organismal development and, in cancer, its mis-regulation is an important component of tumour metastasis. IMP1 function relies on the recognition of a diverse set of mRNA targets that is mediated by the combinatorial action of multiple RNA-binding domains. Here, we dissect the structure and RNA-binding properties of two key RNA-binding domains of IMP1, KH1 and KH2, and we build a kinetic model for the recognition of RNA targets. Our data and model explain how the two domains are organized as an intermolecular pseudo-dimer and that the important role they play in mRNA target recognition is underpinned by the high RNA-binding affinity and fast kinetics of this KH1KH2–RNA recognition unit. Importantly, the high-affinity RNA-binding by KH1KH2 is achieved by an inter-domain coupling 50-fold stronger than that existing in a second pseudo-dimer in the protein, KH3KH4. The presence of this strong coupling supports a role of RNA re-modelling in IMP1 recognition of known cancer targets.

## INTRODUCTION

IGF2 mRNA-binding protein 1 (IMP1) is a conserved RNA-binding protein that plays a key role in regulating cell motility, morphology and differentiation in the embryo, reviewed by Yisraeli (1). The dis-regulation of IMP1 or the expression of non-functional protein leads to impaired embryonic development and pre-natal or neonatal death and in developing neurons, where its function is best studied, IMP1 regulates synaptic morphology and axon outgrowth (2,3). In adults, IMP1 expression is restricted to a small number of tissues and cells (e.g. in the gonads). However, increased expression of IMP1 in cancer cells is related to tumour cell invasion and metastasis, and IMP1 is an important risk factor in cancer relapse (4).

At the molecular level, IMP1 regulates the transport, translation and stability of a diverse ensemble of messenger RNAs (mRNAs). In neurons, the best-studied function of IMP1 is its role in mediating the transport and controlled translation of β-actin mRNA (5,6). IMP1 associates with β-actin mRNA in the perinuclear region (7) and mediates its transport to different axonal and dendritic locations in a translationally repressed state until, in response to signalling, IMP1 dissociates from the mRNA. IMP1 dissociation facilitates mRNA translation (8), and it has been suggested that this is mediated by an ‘unpacking’ of the mRNA target (7). Importantly, IMP1 is also part of the c-myc-Let-7-Lin28 network, which regulates stem cell status and is mis-regulated in a large proportion of cancers (4). Indeed IMP1 increases the stability of the mRNAs encoding the oncoprotein c-Myc (9,10) and the cell-surface glycoprotein CD44 (11) amongst others, and is itself down-regulated by Let-7 miRNA (12). More recent studies have also shown a role for IMP1 in the stabilization of numerous non-coding RNAs (13).

**Figure 1.**
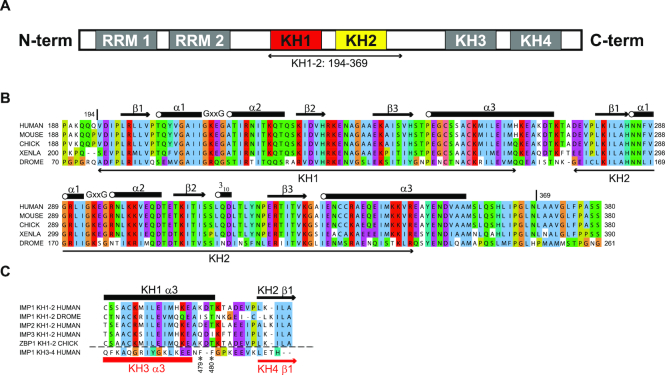
Domain organization of IMP1. (**A**) Schematic representation of the IMP1 protein. The arrow indicates the boundaries of the KH1KH2 construct used in structural studies. (**B**) Sequence alignment of IMP1 KH1 and KH2 from different species. The secondary structure elements derived from the crystal structure are shown in cartoon above the alignment. The arrows below the alignment highlight the canonical KH-domain boundaries. (**C**) Enlargement of the alignment in the KH1KH2 inter-domain linker region. In addition to KH1KH2 sequences, the same region of KH3KH4, aligned to the pre-existing KH1KH2 alignment, is also displayed. The secondary structure elements in KH1 and KH2 are shown in black cartoon above the alignment. The secondary structure elements of KH3 and KH4 are shown in red cartoon below the alignment. F479 and F480 in the KH3KH4 linker are indicated by the asterisks.

IMP1 contains six putative RNA-binding domains—two RNA-recognition motifs (RRMs) and four K-homology (KH) domains (Figure 1A)—which are conserved across species (Figure 1B) and found in pairs (RRM1RRM2, KH1KH2 and KH3KH4) that are closely spaced in the protein sequence (1). Functional and biochemical data from a small number of well characterized mRNAs (β-actin, CD44, c-myc, IGF2) indicate that recognition of the mRNA targets relies on contributions from multiple KH domains (14–16). However, the contribution to binding of the KH domains is target-dependent. In particular, recognition of the CD44 and c-myc 3′UTR (16) requires all four KH domains of IMP1 (11,15), while only the C-terminal KH domains (KH3 and KH4) are required for the recognition of β-actin and a number of other neuronal mRNAs (14). Interestingly, a recent study has shown that in cancer cells, IMP1-mediated regulation of mRNA stability of a number of targets is controlled by m6A methylation of the cognate RNA sequences, and that this effect is linked to the KH3KH4 di-domain (17).

The RNA recognition properties of the KH3KH4 di-domain and its interaction with the β-actin RNA target have been extensively characterized (14,18–20). The sequence targets of the individual KH3 and KH4 domains have been defined and results from different groups have shown that the di-domain binds a bi-partite sequence on the RNA target, re-modelling its structure through RNA looping (14,19). However, it is unknown how the KH1 and KH2 domains interact with RNA and it has been challenging to define a role for these domains in the recognition of functional targets at the molecular level. Importantly, this has also prevented a motif-driven analysis of the transcriptome-wide RNAs bound by IMP1, for example extracted from CLIP data, which could capture the different combinatorial binding modes of the protein. Here, we set out to define the structure and RNA-binding properties of the KH1KH2 di-domain and create a kinetic model of RNA binding that can be used to build a mechanistic understanding of the IMP1–RNA interaction.

## MATERIALS AND METHODS

### Cloning, expression and protein purification

The KH1KH2 di-domain (V194-N369) from human IMP1 (Uniprot Q9NZI8) was expressed and purified in a similar way to that described by Nicastro *et al.* (19). Briefly, ^15^N or ^15^N,^13^C-labelled samples for nuclear magnetic resonance (NMR) were obtained by expressing KH1KH2 as a N-terminal 6xHis-GST-KH1KH2 fusion protein in *Escherichia coli* BL21 (DE3) cells (Invitrogen) grown in M9 minimal media supplemented with ^15^NH_4_Cl and ^12^C or ^13^C-glucose (as required) as the sole nitrogen and carbon sources, while unlabelled samples were obtained by expressing the protein in LB media. The protein was initially purified by immobilised metal affinity chromatography (IMAC) with a Ni-NTA (nitrilotriacetic acid) agarose matrix (ThermoFisher scientific). The His-GST fusion tag was then removed by cleavage with TEV protease and KH1KH2 further purified using a cation-exchange HiLoad SP-Sepharose 26/10 column and then dialyzed into the final NMR buffer (10 mM sodium phosphate (pH 7.4), 50 mM NaCl, 0.5 mM TCEP (tris(2-carboxyethyl)phosphine)). For crystallization and biolayer interferometry (BLI), unlabelled protein was obtained from cells grown in LB media and purified as above with the addition of a final size exclusion chromatography purification step using a HiLoad Superdex75 16/600 column. Protein concentration was determined from the absorbance of the sample at 280 nm and extinction coefficients calculated from the Tyr content of the sequence. In addition to the wild-type protein, two GDDG mutants (KH1DDKH2; K213D, E214D and KH1KH2DD; K294D, E295D) were prepared using a QuickChange II Site-directed Mutagenesis Kit (Agilent), sequence-verified by Sanger sequencing (by Source BioScience, UK), and expressed and purified as above.

### RNA

RNA oligonucleotides were purchased from Dharmacon (GE healthcare), de-protected according to manufacturer’s protocol and lyophilized. The RNA was re-suspended in either nuclease-free water or ITC (isothermal titration calorimetry) buffer containing 40–100 u ml^−1^ (RNAsin, Promega) and the concentration determined by measuring absorbance at 260 nm and extinction coefficients calculated from the base composition of the sequence.

### NMR spectroscopy

NMR experiments were recorded at 37°C on Bruker Avance spectrometers operating at 600-, 700-, 800- or 950-MHz ^1^H frequency. All NMR spectra were transformed using NMRpipe (21) and analysed using CCPN software (22). Protein backbone resonances were assigned using HNCO, HN(CA)CO, C(CO)NH, CBCA(CO)NH and HNCACB experiments (23), while partial assignment of side chain resonances and the NOE cross peaks, used to validate the KH1KH2 inter-domain arrangement, were obtained from 15N-HSQC-TOCSY, 15N-HSQC-NOESY, HC(CO)NH and 13C-NOESY spectra (24).

^15^N *T*_1_ and *T*_2_ values of the backbone amide resonances were obtained from standard relaxation experiments recorded at 800 MHz. Delays were 0.01; 0.05; 0.1; 0.2; 0.4; 0.7; 1.0 and 1.5 s for the *T*_1_ experiments and 0.008; 0.016; 0.032; 0.064; 0.096; 0.12 and 0.16 s for the *T*_2_ experiments (25). The rotational correlation time per residue (*τ*_c_) of IMP1 KH1KH2 was estimated from the *T*_1_/*T*_2_ ratio of residues L200-E352 as described previously, where residues with overlapping resonances were excluded as they could not be fitted reliably. The order parameter (S^2^) per residue was estimated using a model-free analysis, performed in the program TENSOR assuming an overall isotropic motion (26). Heteronuclear NOE values were obtained from standard experiments (25).

Residual dipolar couplings (RDCs) were extracted by In-Phase and Anti-Phase (IPAP) experiments (27) recorded on a 0.3 mM sample of ^15^N-labelled KH1KH2 in NMR buffer with and without filamentous phage Pf1 (ASLA Biotech Ltd, Latvia). RDCs values were obtained by subtracting the reference value in isotropic solution from the values in anisotropic conditions. The experimental dipolar couplings for the individual amides were then compared to RDCs back calculated from the crystal structure of KH1KH2 using the program Module (28).

### Sequence alignments

Sequence alignments were made using the T-Coffee multiple sequence alignment program (29) (accessible at http://www.ebi.ac.uk/Tools/msa/tcoffee/). Alignment figures were generated by using Jalview / ClustalX.

### NMR studies of protein–RNA interactions

Titrations with RNA oligonucleotides were performed by addition of small volumes of highly concentrated RNA(s) (1–4 mM) to 50–75 μM ^15^N-labelled protein samples in NMR buffer supplemented with 40–100 u ml^−1^ (RNAsin, Promega). The pH was monitored to ensure that it remained within ± 0.1 pH unit of the initial condition. ^15^N-SOFAST-HSQC spectra were recorded at each point of the titration, and the chemical shift changes of amide resonances in fast exchange were measured and the reported weighted-average values of ^15^N and ^1^H chemical shift changes given by Equation (1)

(1) \begin{equation*}{\rm{\Delta }}{{\rm{\delta }}_{{\rm{avg}}}}{\rm{ = }}\,{\left( {{{\left[ {{\rm{\Delta }}{{\rm{\delta }}^{\rm{1}}}{\rm{H}}} \right]}^{\rm{2}}}{\rm{ + }}\,{{\left[ {{\rm{\Delta }}{{\rm{\delta }}^{{\rm{15}}}}{\rm{N}}} \right]}^{\rm{2}}}{\rm{/10}}} \right)^{{\rm{1/2}}}}\end{equation*}

The nucleobase preference of the two mutants in each of the four positions of the core sequence recognized by KH domains was assessed using scaffold independent analysis (30) (SIA). SIA experiments were performed on 50 μM samples of IMP1 KH1DDKH2 or 75 μM IMP1 KH1KH2DD to which were added SIA quasi-degenerated RNA pools at a ratio of 1:2. NMR analysis of the free and RNA-bound samples was performed using 2D ^15^N-SOFAST-HSQC experiments in a semi-automated fashion (31). Briefly, samples were stored in 3 mm NMR tubes at 4°C within a Bruker SampleJet auto-sampler and loaded automatically after a short pre-heating 25°C step. Locking, tuning, matching and shimming were performed automatically. For experiments examining each RNA position, the NMR spectra were processed as a pseudo-3D dataset using NMRpipe. The changes in 13 peaks in fast exchange during the titration with RNA were used to extract SIA values (30). Briefly, for each peak the free-to-bound shift was measured for the individual oligo pools. Then, the group of four pools with (A, C, U, G) permutations in one position of the bound sequence were used to obtain the comparative semi-quantitative assessment of the protein nucleobase preference in that position as follows. The free-to-bound shift of each peak was normalized to the higher shift within the four permutations. This provides a comparative ranking per peak, and attributes the same weight to each peak. Then, the normalized values were averaged across the 13 peaks. The resulting SIA scores represent the relative nucleobase preference of the protein in each of the bound positions.

### Crystallization and Structure determination

KH1KH2 was crystallized using sitting drop vapour diffusion. Typically, a 6.2 mg ml^−1^ solution of KH1KH2 in 10 mM sodium phosphate, 50 mM NaCl, 0.5 mM TCEP pH 7.4 was mixed in a 1:1 ratio with a mother liquor containing 51.4% PEG1000, 150 mM MOPS (3-(N-morpholino)propanesulfonic acid) (pH 7.0), 60 mM NaI and 4% acetonitrile. Drops were equilibrated with a reservoir of the crystallization solution in a sealed well of a 96-well plate at 18°C. Crystals appeared within 2–3 days and were further optimized by microseeding under the same conditions. Crystals were harvested by transferring into fresh crystallization solution supplemented with 20% (v/v) glycerol and flash-frozen in liquid nitrogen. The crystals belong to the spacegroup P2_1_ with one copy of KH1KH2 in the asymmetric unit (AU). X-ray diffraction data were collected at 100 K at the I02 beamline at Diamond Light Source (Didcot, UK) and the data processed using the XDS software suite (32). The structure was solved by molecular replacement with PHASER (33) using the Nova1 KH1KH2 di-domain (PDB ID: 2ANR) as a search model. After initial placement, the model was completed by iterative rounds of model rebuilding in COOT (34) and reciprocal space refinement using REFMAC. TLS (Translation/Libration/Screw) groups were calculated using TLSMD (35,36) and used in the final round of refinement.

The final model comprises all residues from 194–362 with the exception of 238 and 239 in the loop that connects β2 and β3 of KH1 and was refined to a *R*_work_/*R*_free_ of 18.1/24.6. The model has good geometry as determined by PROCHECK (37) with 94.0% of residues in the preferred region of the Ramachandran plot, only 6.0% in the additionally allowed region and no outliers. Details of crystal parameters, data collection and structure refinement statistics are presented in Table 1.

**Table 1.** X-ray data collection and structure refinement statistics

	IMP1 KH1KH2
**Data collection**	
Space group	P2_1_
Wavelength (Å)	0.97949
Cell dimensions	
*a, b, c* (Å)	41.42, 32.99, 58.72
*α, β, γ* (°)	90.0, 103.14, 90.0
Monomer/AU	1
Resolution (Å)	37.21–2.20 (2.26)
*R*_meas_ (%)	11.3 (71.9)
Total reflections	24532 (3886)
Unique reflections	8950 (1423)
*I* / σ(*I)*	7.28 (1.89)
CC(1/2)	0.993 (0.607)
Completeness (%)	97.0 (96.8)
Redundancy	2.74 (2.73)
**Refinement**	
Resolution (Å)	37.21–2.20
No. reflections	7377
*R*_work_ / *R*_free_ (%)	18.1 / 24.6
No. atoms	
Protein	1292
Ligand CCN	3
Ligand PO4	10
Water	50
B-factors	
Wilson	34.5
Average	43.0
Protein	42.9
Ligand CCN	54.4
Ligand PO4	52.7
Water	49.7
R.m.s. deviations	
Bond lengths (Å)	0.0135
Bond angles (°)	1.866
Chiral volume (Å3)	0.0832

*Values in parentheses are for highest-resolution shell.

### ITC

All ITC experiments were recorded at 25°C using a MicroCal ITC200 calorimeter (Malvern Instruments) and data were fitted using the Origin 7 package. ITC titrations were performed by successive injections of 2 μl of RNA solution, 0.5 mM for KH1KH2DD titrations or 1 mM for KH1DDKH2 titrations into a cell containing 50 μM of IMP1 KH1DDKH2 or IMP1 KH1KH2DD in 10 mM sodium phosphate (pH 7.4), 50 mM NaCl, 2 mM TCEP and 40–100 u ml^−1^ (RNAsin, Promega). The equilibrium dissociation constant was determined from each titration series by fitting the measured heat of reaction using a 1:1 binding model, as only one KH domain is capable of binding RNA in the constructs used. The heat of dilution of each RNA oligonucleotide was determined by titration into ITC buffer without protein and was subtracted from each corresponding titration curve prior to fitting the data.

### BLI

Bio-layer interferometry (BLI) experiments were performed at 30°C in 10 mM sodium phosphate (pH 7.4), 50 mM NaCl, 0.5 mM TCEP, with 0.5 mg ml^−1^ bovine serum albumin and RNAse inhibitor 40–100 u ml^−1^ (RNAsin, Promega). Experiments were recorded using a ForteBio OctetRed 96 instrument. 5′-Biotinylated c-myc mRNA-derived RNA (0.5 ng μl^−1^) was immobilized on Streptavidin-coated biosensors and incubated with varying concentrations (1–0.03 μM) of wtKH1KH2, or KH1DDKH2 or KH1KH2DD mutants. Equilibrium dissociation constants for RNA–protein interactions were determined from plots of the observed association rate constant (*k*_obs_) as a function of protein concentration (38) using in-house developed software (39).

### Kinetic modelling and calculations

#### Definitions

*k*_on_, *k*_off_ and *K*_d_ are the association rate constant, dissociation rate constant and equilibrium dissociation constant for the binding of KH1KH2. Our BLI data determine these values as 1.01 (± 0.054) × 10^6^ M^−1^s^−1^, 0.047 (± 0.008) s^−1^ and 46 (± 8) × 10^−9^ M, respectively.

*k*_on_1, *k*_off_1 and *K*_d_1 are the association rate constant, dissociation rate constant and equilibrium dissociation constant for the binding of KH1KH2DD. Our BLI data determine these values as 2.74 (± 0.03) × 10^5^ M^−1^s^−1^, 0.483 (± 0.016) s^−1^ and 1.76 (± 0.206) × 10^−6^ M, respectively.

*k*_on_2, *k*_off_2 and *K*_d_2 are the association rate constant, dissociation rate constant and equilibrium dissociation constant for the binding of KH1DDKH2. These values are not available from the BLI experiments.

*k*C1 is the forward rate for the ring closure step, which follows after the binding of KH2 and *k*O1 is rate for the ring opening step, where KH1 dissociates whilst KH2 remains bound. As outlined in Nicastro *et al.* (19), we have assumed that *k*O1 is the same as the dissociation rate of KH1KH2DD, i.e. *k*_off_1 (0.483 s^−1^).

*k*C2 is the ring closure step, which follows after the binding of KH1 and *k*O2 is the ring opening step, where KH2 dissociates whilst KH1 remains bound. As outlined in Nicastro *et al.* (19), we have assumed that *k*O2 is the same as the dissociation rate of KH1DDKH2, i.e. *k*_off_2.

#### Initial estimate of k_on_2, k_off_2 and K_d_2

While we were not able to directly derive values for these three constants from BLI experiments, we can define an approximate range for *K*_d_2 based on our ITC experiments, structural considerations and the diffusion limit. In ITC experiments, we measured values between 50 × 10^−6^ M and 100 × 10^−6^ M for the equilibrium dissociation constant for the interaction of KH1DDKH2 with different RNAs. We have therefore made calculations for *K*_d_2 values of 50 × 10^−6^ M, 100 × 10^−6^ M and 150 × 10^−6^ M.

For *k*_on_2, we made calculations for a range of values in the same order of magnitude as *k*_on_1, (2.74 × 10^5^ M^−1^s^−1^) as the domains are structurally very similar. The values we used were 2.74 × 10^5^ M^−1^s^−1^ / 3 ( = 9.1 × 10^4^ M^−1^s^−1^), 2.74 × 10^5^ M^−1^s^−1^ and 2.74 × 10^5^ M^−1^s^−1^ × 3 ( = 8.22 × 10^5^ M^−1^s^−1^).

The calculations below were performed for the nine possible combinations of *K*_d_2 and *k*_on_2 (with the appropriate value for *k*_off_2 calculated as *k*_off_2 = *K*_d_2 × *k*_on_2) in order to find a parameter-set that reproduced the kinetics of binding of the KH1KH2 wild-type.

### Calculations and validation

Each of the two possible binding pathways for formation of the closed complex (KH1KH2–RNA) is a reversible bimolecular interaction followed by a conformational change. Therefore, consider first the pathway for formation of the closed complex in which the KH1 domain attaches first (see Figure 6).

\begin{equation*}P + R\, \mathop{\rightleftarrows}\limits^{k_{\rm on}1}_{k_{\rm off}1}\,\,\,\,{PR}\,\,\,\mathop{\rightleftarrows}\limits^{k{\rm C}2}_{k{\rm O}2}\,\,\,\,\,{PR^*}\end{equation*}

Because we have assumed that *k*O2 is equal to *k*_off_2 (see above) a value for *k*C2 can be calculated using:

\begin{equation*}{{k{\rm {\rm C}}2}}\,{\rm{ = }}\,{{{k}}_{{\rm{off}}}}{\rm{2}}\,{\rm{x}}\,\left( {{{{K}}_{{\rm{d1}}}}{\rm{ - }}{{{K}}_{\rm{d}}}} \right)\,{\rm{/}}\,{{{K}}_{\rm{d}}}\end{equation*}

Initially, we calculated *k*C2 values for each of the nine combinations of *K*_d_2 and *k*_off_2 (Supplementary Table S1). These values range from ∼170 to 4600 s^−1^ (see Supplementary Table S1). Since all the constants are now known or estimated one can also calculate the values of *k*C1 in the same way. Values of *k*C1 for different *K*_d_2 are reported in Supplementary Table 2.

All combinations of constants should give the *K*_d_ for formation of the complex with KH1KH2 as they were derived from this value. Therefore, it was possible to check which combinations gave a *k*_off_ value that matches the value observed by BLI (0.047 s^−1^). This value depends on the value of *k*_on_2, but not on the value of *K*_d_2. The tested *k*_on_2 values were 9.1 × 10^4^ M^−1^s^−1^, 2.74 × 10^5^ M^−1^s^−1^ and 8.22 × 10^5^ M^−1^s^−1^, as discussed above, and the resulting *k*_off_ values were respectively 0.017, 0.026 and 0.049 s^−1^. As the value of the observed *k*_off_ is 0.047 s^−1^, a *k*_on_2 value of 8.22 × 10^5^ with a *K*_d_2 value of 100 × 10^−6^ M adequately reproduces our data.

Next, we wanted to establish whether 8.22 × 10^5^ M^−1^s^−1^ is the best *k*_on_2 value to reproduce the data and calculate an interval of confidence. We therefore performed simulations using *k*_on_2 values on either side of 8.22 × 10^5^ M^−1^s^−1^, i.e. 5, 6, 7, 8, 9, 10, 11 × 10^5^ M^−1^s^−1^ and tested how the *k*_obs_ and *k*_off_ we have calculated compared with those obtained by BLI (0.301 ± 0.02 s^−1^ for 0.25 μM added protein and 0.047 ± 0.008 s^1^, respectively). This comparison (Supplementary Figure S1) confirms that the experimental data are best reproduced by a *k*_on_2 value ∼8 × 10^5^ M^−1^s^−1^ and that the range of acceptable values is ± 1 × 10^5^ for *k*_obs_ and ± 2 × 10^5^ for *k*_off_. Therefore, we used a *k*_on_2 value of 8 ± 2 × 10^5^ M^−1^s^−1^ and the resulting *k*_off_2, *k*C1 and *k*C2 values in our kinetic model. A similar strategy cannot be used for *K*_d_2. This is because changing *K*_d_2 with a fixed *k*_on_2 has no effect on the association rate at fixed added protein concentration (*k*_obs_) or on the dissociation rate constant (*k*_off_).

Regardless, the latter values and corresponding errors can be calculated as follows:

\begin{equation*}{{kC1}}\,{\rm{ = }}\,{{{k}}_{{\rm{off}}}}{\rm{1}}\,{\rm{x}}\,\left( {{{{K}}_{{d}}}{\rm{2 - }}{{{K}}_{\rm{d}}}} \right)\,{\rm{/}}\,{{{K}}_{\rm{d}}}\end{equation*}

*K*_d_2 (50 × 10^−6^, 100 × 10^−6^, 150 × 10^−6^ M) is much *>K*_d_

Therefore *k*C1 = *K*_d_2 x *k*_off_1 / *K*_d_

Then, the propagation of errors results in *k*_off_1 / *K*_d_ = 10.5 ± 1.85 so that

\begin{equation*}{{kC1}}\,{\rm{ = }}\,{{{K}}_{\rm{d}}}{\rm{2}} \times {\rm{10}}{\rm{.5}}\,{\rm{ \pm }}\,{\rm{1}}{\rm{.85}}\,{{\rm{s}}^{{\rm{ - 1}}}}\end{equation*}

If we use *K*_d_2 = 100 × 10^−6^ M then *k*C1 = 1050 +/- 185 s^−1^

\begin{equation*}{\rm{Also,}}\,{{k{\rm C}2}}\,{\rm{ = }}\,{{{k}}_{{\rm{off}}}}{\rm{2}}\,{\rm{x}}\,\left( {{{{K}}_{\rm{d}}}{\rm{1 - }}{{{K}}_{\rm{d}}}} \right)\,{\rm{/}}\,{{{K}}_{\rm{d}}}\end{equation*}

In this case, the propagation of errors gives (*K*_d_1-*K*_d_) / *K*_d_ = 37.3 ± 7.9

So that *k*C2 = *k*_off_2 × 37.3 ± 7.9 s^−1^

If we use *K*_d_2 = 100 × 10^−6^ M and *k*_on_2 = 8 × 10^5^ M^−1^s^−1^ (see above) then *k*_off_2 = 80 s^−1^ and *k*C2 = 2985 ± 632 s^−1^.

These calculated and BLI-derived values (reported in the ‘Results’ section and figures) have been rounded for both a clearer presentation and to be more representative of the experimental precision of measurements.

### Simulations

Simulations were performed by numerical integration of the system of ordinary differential equations associated with the model presented here. We used in-house software employing the fourth-order Runge–Kutta method as described in (19). The computer code is available upon request.

## RESULTS

### KH1 and KH2 interact to create a stable structural unit for RNA recognition

**Figure 2.**
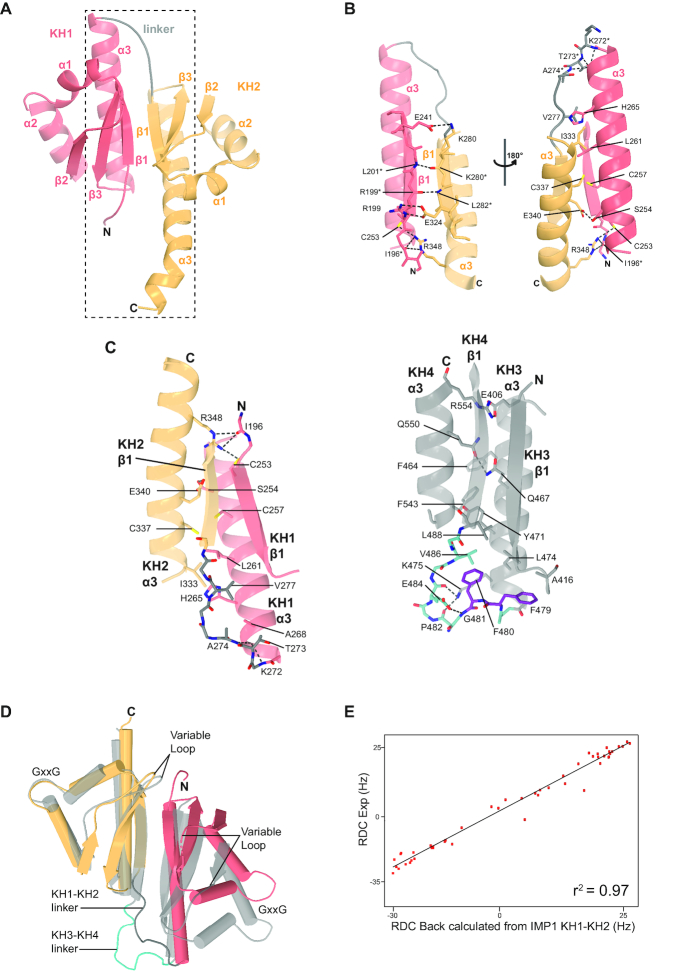
KH1KH2 structure. (**A**) Structure of the KH1KH2 di-domain pseudo-dimer shown in cartoon representation, KH1 is coloured salmon, KH2 in wheat and the inter-domain linker in grey. The N- and C-termini and secondary structure elements are labelled sequentially. (**B**) Close up view of the KH1KH2 interface, boxed in panel (A). Residues that make interactions between the two α3 helices and the linker are shown in stick representation, colour coded by atom type. Hydrogen bonds are shown as dashed lines. (**C**) Comparison of the inter-domain linker and α3 regions for KH1KH2 (left) and for KH3KH4 (right). KH1 and KH2 are coloured as in panel (A) KH3 and KH4 are coloured grey with the KH3KH4 linker in cyan. Residues that make inter-domain contacts or contribute to the hydrophobic cluster in the KH3KH4 structure are shown as sticks, coloured by atom type. F479 and F480 are highlighted in purple. Hydrogen bonds are displayed as dashed lines. (**D**) Structural comparison of the KH1KH2 and KH3KH4 structures. The structures are superimposed by alignment of KH2 and KH4. The KH1KH2 structure is colour-coded as in panel (A) and the KH3KH4 structure is coloured as in panel (C). The GxxG loop, linker and variable loops are labelled. (**E**) The values of the experimentally derived RDC values of the ^1^H-^15^N backbone amides are plotted against the RDC values back-calculated from the KH1KH2 structure using the program Module. The degree of similarity between the measured and calculated values is given by the correlation coefficient R^2^, shown inset.

As a first step to understand RNA recognition by KH1 and KH2, we wanted to address the relationship between the two domains. The two carboxy-terminal domains of IMP1, KH3 and KH4, assemble to form a quasi-symmetric pseudo-dimer (18). However, the two key aromatic residues essential for inter-domain packing, F479 and F480, are not conserved in KH1KH2. (Figure 1C). In order to understand if and how the KH1 and KH2 domains also fold into a pseudo-dimer structure, we expressed an IMP1 construct that includes both domains (residues V194-N369, Figure 1A) and determined the crystal structure. The structure was solved by molecular replacement using the Nova-1 KH1KH2 di-domain (PDB ID: 2ANR) as search model, details of the structure solution and refinement statistics are presented in Table 1. The structure shows that the KH1 and KH2 domains fold into an intra-molecular pseudo-dimer, (Figure 2A), an arrangement found in a number of KH and RRM-containing proteins including the IMP1 KH3KH4 di-domain. Within the pseudo-dimer, the domains make contact through the two β1 strands, the inter-domain linker and the C-terminal α-helix of each domain (α3) (Figure 2B), burying a surface of ∼1200 Å^2^. Interestingly, many of the amino acids mediating the contacts between the two α3 helices (S254, C257, H265) are conserved in KH1KH2 across species but are very different in KH3KH4 (Figures 1C and 2C). In addition, KH1 α3 is longer than KH3 α3 and the linker between KH1 and KH2 is three amino acids shorter than that between KH3 and KH4, implying that the shorter KH1KH2 linker must span a longer distance to reach the amino-terminus of the second KH domain. This is achieved by taking a more direct route in connecting the two domains. In KH1KH2, after a sharp downward turn, the linker assumes a nearly linear conformation retracing the direction of the helix to reach the amino-terminal residue of KH2. In contrast, in the KH3KH4 di-domain the longer linker takes a less direct route and creates a small hydrophobic cluster around the two conserved phenylalanine side chains. (Figure 2C). The differences in the inter-domain interface result in a change in the angle between the two domains and, as a consequence, in a shorter distance (7–8Å) between the two RNA-binding grooves, as highlighted by the superimposition of the two structures (Figure 2D). The structure also shows that the path between the RNA-binding grooves of KH1 and KH2 is positively charged (Supplementary Figure S2), which may impact on the dynamics of RNA looping as discussed in our model below. In summary, the crystal structure indicates that the KH1 and KH2 domains form a pseudo-dimer with an inter-domain arrangement similar to that of KH3KH4 (18). However, localized and absolutely conserved differences at the inter-domain interface, result in a change in the relative position of the RNA-binding surfaces and, potentially, a change in the coupling of the RNA-binding activities of the domains. In addition, the α3 of KH2 is extended by three turns. The extension does not make contacts with other structural elements in KH2 or KH1 (Figure 2A), yet its sequence is highly conserved across species (Figure 1B), indicating a functional role is likely. It seems possible this role is related to RNA binding, as the carboxy-terminal end of α3 makes sequence independent contacts with the bound RNA in a number of KH–RNA structures, for example the one of Nova-2 KH3–RNA (40).

To validate the key contacts between KH1 and KH2 structural elements, we used NMR spectroscopy and assess whether the inter-domain orientation of KH1 and KH2 in the crystal structure corresponds to the conformation in solution. First, using diagnostic NOE cross-peaks from ^13^C-edited and ^15^N-edited 3D NOESY spectra, we confirmed the β1–β1 and α3–α3 interactions and the conformation of the linker (Supplementary Figure S3). Then, we used ^1^H-^15^N RDCs to assess whether the orientation of the two domains in the crystal structure represents the main conformation in solution. RDCs report on the orientation of the individual backbone amide NH vectors of a protein in solution. Therefore, we recorded RDCs on a sample of KH1KH2 protein and compared them with RDCs back-calculated from the X-ray structure. A plot of the RDCs measured in solution against those back-calculated from the structure (Figure 2E) reveals the high correlation (*R*^2^ = 0.97) between the two datasets, indicating that the crystal structure is highly representative of the protein conformation in solution.

Finally, we used NMR relaxation experiments to assess the dynamics of the inter-domain contacts and the internal motions observed in the pseudo-dimer. We recorded ^15^N *T*_1_ and *T*_2_, and ^1^H-^15^N NOE experiments and calculated a KH1KH2 rotational correlation time (τ_c_) of 9.8 ± 0.6 ns. This *τ*_c_ is consistent with a globular or quasi-globular protein of ∼20 kDa, the molecular weight of the KH1KH2 di-domain. Also consistent with the structure, the relaxation experiments reported that the inter-domain linker is not flexible but tumbles coherently with the two domains. By contrast, significant motions are observed in the GxxG and variable loops of KH2, which are often flexible in KH domains (41). Resonances from the GxxG loop of KH1 were not visible in our spectra, also consistent with the flexibility of this loop. Overall, the relaxation data indicate that the two domains form a stable monomeric structural unit with flexible GxxG and variable loops (Supplementary Figure S4).

### KH1 and KH2 have different RNA-binding properties and unique sequence specificity

As for other multi-functional RNA regulators, IMP1–RNA recognition is mediated by the protein’s KH domains in a combinatorial and target-specific fashion. The contribution of individual domains is encoded by their intrinsic RNA-binding properties and by their coupling with other domains. Understanding these properties and coupling can help to refine the molecular understanding of recognition and the interpretation of complex patterns from transcriptome-wide studies.

**Figure 3.**
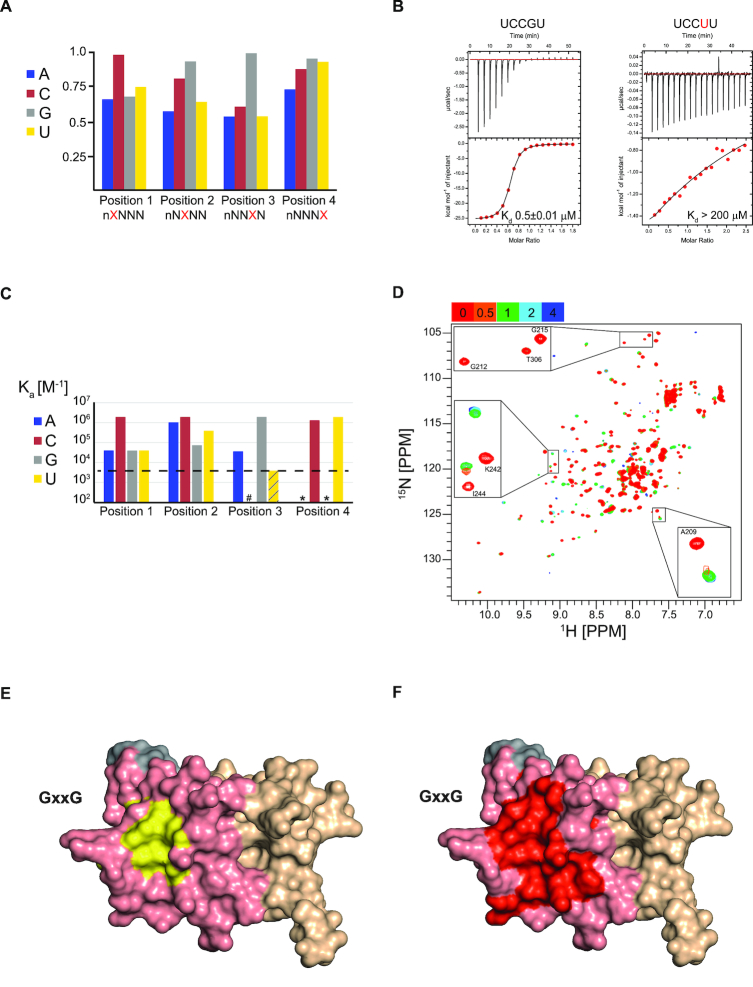
KH1–RNA interaction. (**A**) Bar chart displaying the values of the normalized comparative, semi-quantitative SIA scores (*Y-*axis) indicating the relative preference of KH1 for the four nucleobases (colour coded) in positions 1–4 of the bound RNA oligo (*X-*axis). (**B**) ITC thermograms for the interaction between IMP1 KH1KH2DD with the specific UCCGU RNA target (left) and the variant UCCUU RNA (right). The raw data are shown in the upper panels and binding isotherm in the lower panels. The black line represents the best fit of the data to a single-binding site model, the *K*_d_ values are reported on each plot. (**C**) ITC-derived *K*_a_ values for the KH1KH2DD interaction with single nucleotide permutations of the KH1 consensus sequence, which have been tested to define the nucleobase preference in the four positions of the bound RNA. Position 4 has been tested with only two nucleobases, to validate the lack of specificity. The nucleobases are colour coded as in A; asterisks indicate untested nucleobases. RNAs with *K*_a_ lower than the black broken line bind to weakly for the measured affinity to be considered reliable, indicated with a # for the C permutation of position 3 where no binding is visible, and a dashed box reaching the boundary, for the U permutation in position three where a *K*_d_ > 200 μM was calculated. (**D**) ^15^N-^1^H-correlation spectra recorded during the titration of KH1KH2DD with UCCGU. The colour code of the molar ratios is reported in the top left of the spectrum. Most of the peaks with significant chemical shift changes are in the intermediate/slow exchange regime, see insets. (**E**) Molecular surface representation of the KH1KH2 di-domain highlighting the interaction surface. KH1 is coloured in salmon, KH2 in wheat and the inter-domain linker in grey. The hydrophobic residues in the KH1 RNA-binding groove (I, L, V, A and F) are coloured in yellow. (**F**) Molecular surface representation of the KH1KH2 di-domain coloured as in E. The residues in KH1KH2DD with amide chemical shifts that change significantly upon titration of a UCCGU oligo are coloured in red. The location of the KH1 GxxG motif in the structure in E and F is indicated.

In order to de-convolute the RNA-binding properties of the IMP1 KH1 and KH2 domains, we have used site-specific mutations that knock out the RNA-binding activity of KH domains. The individual KH1 and KH2 domains when expressed alone in *E. coli* are weakly expressed and poorly soluble. Therefore, to assess the contribution from KH1, we have used an IMP1 mutant (KH1KH2DD), where the conserved RNA-binding GxxG-loop of KH2 was mutated to GDDG. The two negatively charged residues prevent the interaction between the loop and the backbone of the RNA and abolish RNA binding by KH2. This mutant can then be used to study directly the KH1–RNA interactions in the context of the entire KH1KH2 structural unit. Fingerprint ^1^H-^15^N HSQC NMR experiments and CD-monitored temperature unfolding of the protein confirmed that this double mutation does not disrupt the structure of the domain or cause significant changes in stability (Supplementary Figure S5). To analyse the RNA-binding specificity of KH1 alone in the context of KH1KH2DD, we first performed SIA (30) to obtain an initial assessment of the nucleobase preference at each of four positions in the bound RNA sequence. Most KH-domains bind five nucleotides, which are typically numbered from 0 to 4 in a 5′ to 3′ direction. Of these five nucleobases only the four in positions 1–4 are accommodated in the RNA-binding groove and are recognized with different degrees of affinity and specificity in different KH domains. Therefore, SIA assays were used to test the comparative nucleobase preference in positions 1–4 of a KH1-bound sequence in the KH1KH2DD di-domain. The results (Figure 3A) indicate that KH1 recognizes with high specificity a C in position 1 and a G in position 3, while it has a lower specificity in position 2, with a C or a G being favoured and in Position 4 no base preference is apparent. In order to validate these data and quantify nucleobase preference, we used ITC to measure the affinity of the four possible permutations (A, C, G, U) for each of the three positions that showed a significant nucleobase preference (positions, 1, 2 and 3). In addition, we tested a single U to C change in position 4, to validate the low of specificity in this position. We based the ITC titration series around a SIA-derived consensus pentamer (UCCGU, positions 0-to-4) chosen to minimize the possibility of inter-molecular RNA duplex formation. The ITC data (Figure 3B and Supplementary Figure S6) reveal the strong specificity of positions 1 and 3. Position 3 is highly selective for G, as replacement of the Guanine with other bases results in a loss of affinity of 25-fold or greater. For example, replacing G with U, results in a >400-fold reduction in the binding affinity (*K*_d_ = 0.5 μM to *K*_d_ > 200 μM). At position 1, replacement of C with any other nucleotide results in around a 20-fold loss of affinity. Overall, the ITC analysis of the binding of all sequence variants displays a trend that mirrors the SIA results (Figure 3A and C), although the affinity for the UCGGU RNA is lower than expected, possibly because of the higher tendency of this short RNA to form base pairs.

To validate our binding model, where KH1 interacts with RNA via the canonical RNA-binding groove, we used NMR Chemical Shift Perturbation (CSP) experiments (Figure 3D–F and Supplementary Figure S7). The titration of KH1KH2DD with the high-affinity UCCGU RNA showed that the RNA interacts with the RNA-binding groove, with minor shifts visible at the carboxy-terminal end of helix 3 (Figure 3E and F). This is similar to what is observed in the Nova-1 KH3–RNA interaction (40) where non-specific contacts take place between the nucleotide in position 0 and the equivalent helix 3. The NMR experiments also confirmed that the domain binds the target sequence with high affinity, as many of the perturbed peaks are in intermediate exchange consistent with a micromolar equilibrium dissociation constant. Interestingly, the sequence specificity of KH1 is unique so far amongst KH domains, with the closest match being KSRP KH3 that also recognizes with high specificity a G in the key position 3. However, unlike IMP1 KH1, KSRP KH3 prefers a G in position 2 and does not show a strong nucleobase preference in position 1 (42). NMR CSP experiments also showed that two residues involved in the KSRP KH3–RNA interaction are conserved in KH1 and are affected upon addition of RNA. It seems possible that KSRP KH3 and IMP1 KH1 share at least some of the features of RNA recognition, possibly including the double hydrogen bond with the amino and carboxyl moieties of two neighbouring amino acids on the second β-strand of the domain.

**Figure 4.**
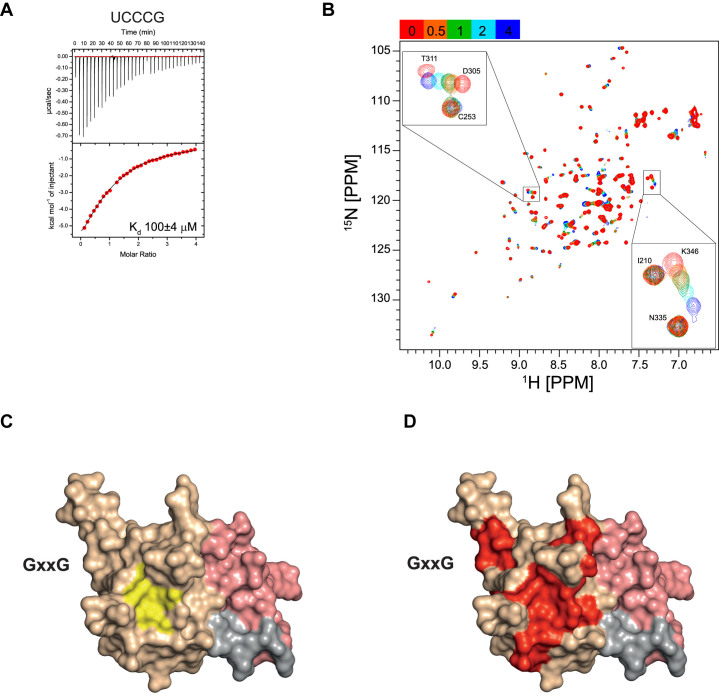
KH2–RNA interaction. (**A**) ITC thermogram of the interaction between IMP1 KH1DDKH2 and the UCCCG RNA. The upper panel is the raw data and the binding isotherm is displayed in the lower panel. The solid line represents the best fit of the data to a single-binding site model. The *K*_d_ value is reported on the plot. (**B**) ^15^N-^1^H-correlation spectra recorded during the titration of KH1DDKH2 with UCCCG. The colour code of the molar ratios is reported in the top left of the spectrum. Most of the peaks with significant chemical shift changes are in the fast exchange regime, indicating weak binding, see insets. (**C**) Molecular surface representation of the KH1KH2 di-domain. KH2 is coloured in wheat and KH1 in salmon. The hydrophobic residues in the KH2–RNA binding groove (I, L, V, A and F) are coloured in yellow. (**D**) Molecular surface representation of the KH1KH2 di-domain coloured as in C. The residues in KH1DDKH2 with amide significantly chemical shifts that change significantly upon titration of the UCCCG oligo are coloured in red. The location of the KH2 GxxG motif in the structure is indicated.

CD and NMR experiments also confirmed that a KH1DDKH2 mutation designed to abolish KH1–RNA binding does not perturb the structure of the domains, nor significantly change their stability (Supplementary Figure S5). This enabled the nucleobase preference of KH2 to also be tested by SIA. In these experiments, low data quality prevented us from reliably measuring chemical shift changes. However, a qualitative comparison of the data from the relevant NMR titrations indicated that an nnCCG sequence was a reasonable starting point for assessing binding specificity. ITC titrations (Figure 4A and Supplementary Figure S8) recorded on the permutation of the four nucleotides (A, C, U, G) in the 2, 3 and 4 positions confirmed that the affinity of KH2 for an RNA pentamer is low (*K*_d_ > 50 μM for all tested pentamers Supplementary Figure S8) and that the domain sequence specificity is weak. While some nucleobases are better tolerated than others in positions 2 and 4 (*K*_d_ between 50 and 150 μM) no binding at all could be measured for other combinations. Therefore, no absolute specificity could be determined in our assays at any of the four positions. Figure 4A shows an example ITC titration with a UCCCG RNA that binds with a measurable affinity (*K*_d_ of 100 μM). As for KH1, NMR CSP mapping of the protein–RNA interactions confirmed that the RNA binds in the canonical RNA-interaction groove of the domain, with minor contacts been made with the carboxy-terminal of α3 (Figure 4B–D and Supplementary Figure S9). However, in contrast to the KH1KH2DD experiment, NMR titrations showed that in KH1DDKH2-RNA binding most residues are in a fast exchange regime, consistent with the weaker KH2–RNA interaction (Figure 4B). Given these unexpected differences in RNA-binding affinity and different degree of sequence specificity displayed by the KH1 and KH2 domains, we next set out to examine how these two domains operate in the structural unit with the objective to define a model for the recognition of KH1KH2–RNA targets.

### KH1 and KH2 collaborate to bind a c-myc-derived RNA

**Figure 5.**
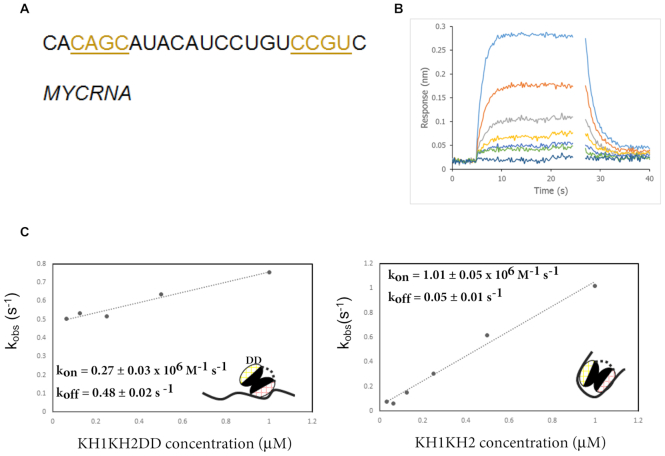
BLI analysis of KH1KH2–RNA binding. (**A**) The MYCRNA sequence, the sequences matching the KH1 consensus are underlined and coloured in gold. (**B**) BLI measurement of IMP1 KH1KH2DD–MYCRNA interaction. Biotin-immobilized MYCRNA was incubated with increasing concentration of IMP1 KH1KH2DD (lower to upper curves). Only the fast association and dissociation phases of the recorded interferograms are displayed that were used to obtain the kinetic parameters for the interaction. A linear slope was subtracted from the raw data in order to correct for a drift in the baseline prior to data analysis. Raw data are presented in Supplementary Figure S10. (**C**) Left, The data from B were used to extract the observed association rate constant *k*_obs_ and are plotted as a function of KH1KH2DD concentration. Right, An equivalent plot for the interaction between wild-type KH1KH2 and MYCRNA. The raw interferograms are presented in in Supplementary Figure S10. Inset are cartoons of the KH1 and KH2 domains drawn as two ellipsoids with the RNA-binding surfaces coloured in pink (KH1) and yellow (KH2). ‘DD’ represents the GDDG mutation. The rounded values of *k*_on_ and *k*_off_ and associated errors are also reported.

The structure of the KH1KH2 di-domain (Figure 2) shows the two domains are physically coupled through an extended interface, which suggests that the binding of KH1 and KH2 to RNA targets may also be coupled. In order to investigate this idea, we wanted to examine the interaction of KH1KH2 and the two GDDG mutants using BLI and an RNA target that contains the binding site of both domains. However, the mapping resolution of the IMP1-binding sites in KH1KH2-dependent IMP1 target RNAs is typically on the scale of hundreds of nucleotides (11,16) and so the precise interaction site of KH1 (or KH2) has not yet been defined in any of the IMP1 cellular targets. In addition, similar to what is observed with KH3KH4, KH1 could in principle bind to RNA-recognition sites located both 5′ or 3′ of KH2 (Figure 5A), and this needs to be considered when choosing the model RNA target. However, as KH2 has very low sequence specificity, an RNA which contains a KH1 recognition sequence at both 5′ and 3′ ends and is sufficiently long to allow binding of both domains (4–5 nucleotides per domain separated by a minimum 11–12 nucleotide-long linker based on the structural comparison with KH3KH4) can bind simultaneously KH1KH2 in either orientation (Figure 5A). One such KH1KH2-binding sequence is present within the c-myc mRNA stability element (CRD element) (9) (Figure 5A–C and Supplementary Figure S10A–C). This binding sequence, which we name MYCRNA (Figure 5A) is also in the proximity of a putative target site for IMP1 KH4 (Supplementary Figure S10A), and was used as a model for our biophysical investigation of the KH1KH2–RNA interaction.

The BLI interferogram series recorded using immobilized MYCRNA and increasing concentrations of KH1KH2DD displayed a fast, concentration dependent, association phase (Figure 5B). We analysed the observed rate constant (*k*_obs_) for the concentration-dependent binding as a function of protein concentration to obtain kinetic constants (*k*_on_1 = 2.74 ± 0.03 × 10^5^ M^−1^s^−1^ and *k*_off_1 = 0.48 ± 0.02 s^−1^) and therefore the affinity (*K*_d_1 = 1.76 ± 0.2 μM) of the KH1–RNA interaction (Figure 5C). This analysis shows that KH1 binds to the RNA with an affinity similar to that of the KH4–RNA and KH3–RNA interaction (*K*_d_ ∼ 1 μM for both domains), while the kinetics of the interaction show that the association and dissociation of the domain are faster than those of KH3 and KH4 (KH3 *k*_on_ ∼ 3 × 10^4^ M^−1^s^−1^, KH4 *k*_on_ ∼ 1.6 × 10^5^ M^−1^s^−1^, KH3 *k*_off_ ∼ 0.046 s^−1^, KH4 *k*_off_ ∼ 0.13 s^−1^). Equivalent experiments were recorded on the KH1DDKH2 mutant to assess KH2 binding, but we could not obtain a clear concentration dependent association, most likely because of the low affinity of this domain for MYCRNA.

Having explored the affinity and kinetics of individual KH1 and KH2 domains, we analysed the interaction between the wild-type protein KH1KH2 and the MYCRNA, where both domains can engage in the interaction. As observed for KH1 binding, the interferograms showed a fast, concentration-dependent association whose analysis yielded a *k*_on_ = 1.01 ± 0.05 × 10^6^ M^−1^s^−1^ and a *k*_off_ = 0.05 ± 0.01 s^−1^, resulting in a *K*_d_ of 46 ± 8 nM (Figure 5C and Supplementary Figure S10C). This represents an increase of ∼40 fold over the binding of the high-affinity KH1 domain and indicates that binding of the two domains is coupled. Whilst this coupling is not strong in absolute terms, (0.025, from *K*_a_/ *K*_a_1 ×*K*_a_2), as is typically observed in multi-domain RNA recognition, it is however nearly two orders of magnitude stronger than that observed in similar di-domain units, as discussed below. While no values are reported in the literature for the affinity of KH1, KH2 or KH1KH2 for RNA, a full-length IMP1 protein with mutations that impair RNA binding in KH3 and KH4 has been reported to bind RNA with a *K*_d_ ∼ 60 nM (16). Our study reveals that the RNA-binding affinity of isolated KH1KH2 is high, similar to that of KH3KH4 and that binding of KH1 and KH2 is coupled. Interestingly, the increase in IMP1-binding affinity we observed as a consequence of binding both domains, stems both from a faster association rate constant (∼1 × 10^6^ M^−1^s^−1^) with the RNA and a slower dissociation rate constant (∼0.05 s^−1^).

### Fast RNA looping kinetics couple KH1–RNA and KH2–RNA binding to create a tight KH1KH2–RNA complex

**Figure 6.**
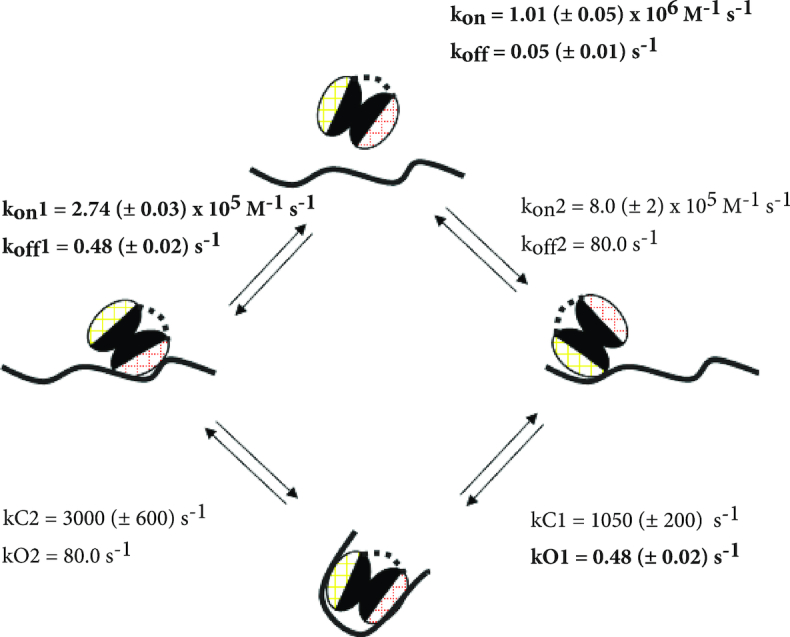
Kinetic model for the KH1KH2–RNA interaction. KH1 and KH2 are represented by two ellipsoids as in Figure 5, with the RNA-binding surfaces in pink (KH1) and yellow (KH2), the RNA is represented by a black line. The rate constants modelled for each step of the process are shown with those derived experimentally by BLI (also shown in Figure 5) in bold.

Next, we employed a kinetic simulation to describe the two-step KH1KH2–RNA interaction in order to provide a mechanistic insight into RNA recognition and re-modelling. In addition, these calculations allowed us to derive a number of important parameters that are not accessible experimentally. In the model, the KH1KH2–RNA interaction is considered as a reversible bi-molecular interaction followed by a concentration-independent conformational change. This can be visualized as the binding of RNA to either KH1 or KH2 followed by looping of the RNA and then a second RNA-binding event at the unfilled site (Figure 6). This mechanism is described by a number of kinetic constants, including the association and dissociation rate constants for the KH1– and KH2–RNA interactions and the ‘closing’ constants *k*C1 and *k*C2, that describe the conformational rearrangements taking place in the two different pathways (Figure 6). The kinetic constants for the binding of wild-type KH1KH2 and KH1KH2DD to the MYCRNA were determined experimentally using BLI. As noted above, the affinity and kinetic constants of KH2–MYCRNA binding could not be measured using BLI and had instead to be estimated (see ‘Materials and Methods’ section) and then validated by comparing calculated values with the observed overall dissociation rate constant (*k*_off_) and the observed association rate at 0.25 μM added protein (*k*_obs_).

In practice, we tested a range of values for the estimated kinetic constants (see ‘Materials and Methods’ section). This test established that a *k*_on_2 = 8.0 ± 2 × 10^5^ M^−1^s^−1^ provides the best fit to the *k*_off_ and *k*_obs_ values discussed above (Supplementary Figure S1). Assuming a *K*_d_2 ∼ 100 μM, this *k*_on_2 corresponds to a *k*_off_2 ∼ 80 s^−1^. Comparison of KH1 and KH2 association rate constants showed that KH2 associates faster with the RNA than KH1. This may be linked to KH2 capacity to interact with multiple neighbouring sequences that provide a high local concentration of sites that can be visited in a series of weak encounters. This is also consistent with the short lifetime of the KH2–RNA complex. It is also notable that the KH1KH2 association rate constant is close to that of KH2, suggesting a leading role for this domain in the initial association between KH1KH2 and RNA. Similarly, comparison of dissociation rate constants showed that the lifetime of the KH2–RNA complex is much shorter than that of the KH1–RNA complex, which accounts for the lower binding affinity of KH2. Importantly, our simulations show that the second concentration-independent step of the reaction, which involves the RNA looping and the binding of the second domain, is very fast and drives the overall reaction. We propose the high values of *k*C1 (1050 ± 200 s^−1^) and *k*C2 (3000 ± 600 s^−1^), that are a result of the strong coupling between domains, compensate for the short lifetime of the KH2–RNA interaction (*k*_off_2 = *k*O2 ∼ 80 s^−1^) and lead to a high-affinity (*K*_d_ ∼ 46 nM) KH1KH2–RNA interaction.

Our simulations require estimates of the protein and RNA concentrations in living cells. The cellular concentration of IMP1 has been quantified in the sub-micromolar range both in a line of highly proliferating cancer cells (K562) (43) and in developing neurons (44). For our simulations, we used a protein concentration of 200 nM as we have done previously for the simulation of the IMP1 KH3–KH4 RNA interaction (19). The c-myc mRNA concentration is highly regulated, but is generally lower by an order of magnitude than that of β-actin mRNA, which has been estimated at 100 pM to 1 nM in most proliferating cells. Therefore, we estimate c-myc mRNA concentration is in the picomolar range (we used a nominal concentration of 200 pM). At these protein and RNA concentrations, given the affinity of the KH1KH2–RNA interaction more than 80% of the RNA target would be bound by IMP1 (Supplementary Table S3). Furthermore, at protein concentrations so far below *K*_d_1 and *K*_d_2 an alternative complex where a single RNA molecule binds two protein molecules cannot form in significant amounts. Finally, at this protein to RNA stoichiometry, the amount of bound RNA depends on the protein, not the RNA concentration. Overall, our calculations describe previously inaccessibly kinetic parameters and provide a mechanistic insight in KH1KH2–RNA binding.

## DISCUSSION

This work explores the structure and RNA-binding properties of the KH1 and KH2 domains of IMP1 and builds a mechanistic model for RNA recognition. IMP1 function is mediated by the recognition of a diverse ensemble of functional RNA targets and the KH domains have target-dependent roles in IMP1–RNA recognition (14–16). Therefore, a first and crucial step in decoding these different roles at the molecular level is to establish whether the RNA-binding domains have intrinsically different RNA-binding properties. Our analysis indicates that this indeed is the case, and we now show that all four KH domains of IMP1 have significantly different specificity and binding kinetics. KH1 shows a novel specificity and significant nucleobase discrimination at position 1 and 3 of the interacting RNA sequence, but only a very limited specificity is observed for KH2. By contrast, KH4 has been reported to recognize specific nucleobases in four of the bound positions, and KH3 in two or three (14,19). Interestingly, the sequences recognized by the individual domains are also very different, CNG for KH1, CA or ACA for KH3 and CGGAC or GGAC for KH4. Arguably, the differences in the level of specificity and in the recognized RNA sequences as well as the high-affinity of the independent di-domain RNA-recognition units—which are sufficient to mediate an efficient interaction at the cellular protein and RNA concentrations, (Supplementary Table S3)—provide a molecular framework for the differential recognition of IMP1–mRNA targets.

At present we have only a rudimentary understanding of which RNA sequences are recognized in IMP1 targets in the transcriptome, as defined by CLIP- and CLIP-associated methods. Different transcriptome-wide analyses of IMP1-binding sites have identified the dinucleotide CA as the most common motif in IMP1 target sequences (13,45,46). In contrast, a recent re-visitation of some of these data has reported that the KH4 consensus sequence, GGAC, is also highly enriched (17). These results highlight that the deconvolution of different motifs *de novo* from the transcriptome-wide binding sites of a protein is challenging. However, prior knowledge of the target sequence provides a tool to separate different binding modes and ascribe functional implications, as recently shown for the cancer factor RBM10 (47). The identification of the consensus sequence for KH1 will likely be invaluable in interrogating transcriptomic data that reports on IMP1–RNA interactions in many cancer cells, cell lines and ES cells.

Also important for the functional decoding of protein–RNA recognition is an understanding of the kinetics of protein–RNA interactions. Our BLI data indicate that the KH1KH2 and KH3KH4 di-domains have very different kinetics of interaction with the RNA. While KH1KH2 binds to a target RNA, an order of magnitude faster than KH3KH4 (*cf*. 1.0 × 10^6^ M^−1^s^−1^ and 1.6 × 10^5^ M^−1^s^−1^), the lifetime of KH3KH4 is ∼14 times longer than that of the KH1KH2–RNA complex (Figure 6 and in Nicastro *et al.* (19)). The low specificity and fast kinetics of KH2 contrasting with the high specificity of KH4 for the RNA hint that this domain may play an early role in IMP1 binding by mediating relatively dynamic and non-specific contacts with the RNA, possibly driven by an avidity effect. If the KH1 target sequence is present, the encounter complexes between KH2 and the RNA would be readily stabilized by the binding of the coupled KH1 domain.

An important consequence of IMP1–RNA binding is the re-modelling of the local RNA structure. The well-described RNA re-modelling by the KH3KH4 di-domains (14,18), together with fluorescence correlation spectroscopy data linking IMP1–mRNA dissociation to a change in RNA mobility (7), indicate that IMP1 function may be associated with re-modelling or ‘packaging’ of the mRNA targets. However, if and how KH1 and KH2 also contribute to the re-modelling of the diverse set of RNA targets was unclear. Our data show that the RNA-binding affinity and the inter-domain coupling of KH1 and KH2 are very different from that of KH3 and KH4, although the arrangement of the di-domain unit is similar in KH3KH4, both KH3 and KH4 bind RNA with *K*_d_ ∼1 μM. In contrast, in KH1KH2 the affinity of KH2 (*K*_d_∼100 μM) is ∼2 orders of magnitude lower than that of KH1 (0.5 μM) and this could influence the RNA-binding mode and its capacity to re-model the RNA. However, we show that, despite the low-binding affinity of KH2, KH1KH2 binds RNA with an overall *K*_d_ in the nanomolar range. Our simulations explain that in the second step of KH1KH2 binding, a very fast ‘closing’ of the RNA loop compensates for the short lifetime of the KH2–RNA interaction. Arguably, one of the determinants of this is the fast association of the KH2 domain with RNA, which is 10 times faster than any of the other three KH domains. Other contributions to overall affinity may derive, for example, from positively charged residues on the protein surface (Supplementary Figure S2), which could direct the RNA chain towards the second RNA-binding groove on KH1 after the initial encounter with KH2. Regardless, these data describe the plasticity of a two-domain system that can accommodate very different affinities and specificities of the two individual domains. In IMP1, this understanding provides a mechanistic framework to interpret the role of KH1KH2 in the interaction with cancer related targets. More broadly, this concept applies to other proteins that contain similar di-domain RNA recognition units, KH or RRM-based, and it explains that RNA re-modelling may be a broader phenomenon than initially thought.

## DATA AVAILABILITY

The IMP1 KH1KH2 coordinates and structure factors have been deposited in the Protein Data Bank under accession number 6QEY. NMR resonance assignments have been deposited in the Biological Magnetic Resonance Data Bank under accession number 27777. The plasmid for the expression of IMP1KH1KH2 is available from the authors upon request. The computer code for the kinetic simulations of IMP1 KH1KH2–RNA interactions is available from the authors upon request.

## Supplementary Material

Supplementary Data
