# Supplementary material for: IMP1 KH1 and KH2 domains create a structural platform with unique RNA recognition and re-modelling properties
Source: Nucleic Acids Res. 2019 Mar 13;47(8):4334–48. doi: 10.1093/nar/gkz136 (PMC6486635; doi:10.1093/nar/gkz136)
Supplement: Supplementary Data [file gkz136_supplemental_file.pdf]

Supplementary Materials

IMP1 KH1-KH2 domains create a structural platform with unique RNA recognition and re-modelling properties

Dagil *et al.*

Supplementary Figures legends

Figure S1: Validation of the $k_{on}2$ value used in our kinetic model. (A) A range of values of $k_{on}2$ (defined as in Methods and in Figure 6) is plotted against the corresponding calculated values of k_{obs}. The experimental value of the KH1KH2 k_{obs} at 0.25 μM added protein (0.3 s^{-1}) is indicated by a dashed line and is best matched by a $k_{on}2$ value of 8 x 10^5 $M^{-1}s^{-1}$. The two dotted lines indicate the acceptable values derived from the error in k_{obs} (± 0.02 s^{-1}), from which a ± 1 x 10^5 $M^{-1}s^{-1}$ error in $k_{on}2$ can be derived. (B) The same values of $k_{on}2$ are plotted against the corresponding calculated values of k_{off}. The experimental value of the KH1KH2 k_{off} (0.047 s^{-1}) is indicated by a dashed line and, as above, the experimental value of k_{obs} fits best to a $k_{on}2$ value of 8 x 10^5 $M^{-1}s^{-1}$. This value will be therefore used in our calculations. The two dotted lines indicate k_{off} (± 0.008 s^{-1}) from which a ± 2 x 10^5 $M^{-1}s^{-1}$ error in $k_{on}2$ can be derived. We have used the larger of two errors in the manuscript.

Figure S2: The KH1KH2 path between the RNA binding grooves is positively charged. Two 180°-rotated surface representations of the KH1KH2 di-domain, colour coded by charge, are shown. The structures are oriented to display the two shortest paths between the RNA binding grooves. Green stars mark the expected positions of the two RNA chains based on our NMR chemical shift mapping and by comparison with the structure of the KH3-KH4-RNA complex.

Figure S3: The inter-domain conformation observed in the crystal structure is validated by diagnostic inter-molecular NOEs. (Left) NOE ^{1}H-^{1}H cross-peaks from interfacing residues in the 3D ^{13}C-edited and ^{15}N-edited NOESY spectra of IMP1 KH1KH2. Top and upper middle, two ^{13}C planes are shown so to display the forward and back NOEs between the L200 Hα and I281 Hα and the H265 Hβ and the I333 Hδ1 respectively. Lower middle, the forward NOE between E276 Hα and H265 Hδ, the back NOE cannot be distinguished clearly. Bottom, the NOE between the amide protons of L201 and K280. (Right), residues responsible for the diagnostic NOEs are highlighted in stick representation on the structure (Top L200-I281, Upper Middle H265-I333, Lower Middle H265-E276 and Bottom L201-K280). The protein backbone is displayed in a cartoon representation, KH1 is coloured salmon, KH2 in wheat and the interdomain linker in grey. The assigned NOEs are represented by the dashed green linking between 1H atoms.

Figure S4: KH1KH2 dynamics (A) Rotational correlation time (Tc) determined for each residue in KH1KH2 plotted against sequence position. (B) Order parameter (S) determined for each residue plotted against sequence position.

Figure S5: Structure and stability of the KH1KH2DD and KH1DDKH2 constructs. (A) Superimposition of the ^{1}H-^{15}N-correlation spectra of the wild type KH1KH2 (black), KH1KH2DD (red) and KH1DDKH2 (blue). Only resonances from residues in close proximity to the mutations show any chemical shift perturbation, indicating the global structure is not changed by the amino-acid substitutions. Linewidths of the three spectra are comparable indicating DD mutations have not caused protein aggregation. (B) The CD signal (220 nm) of the KH1KH2, KH1DDKH2 (red) and KH1KH2DD (blue) proteins recorded during the temperature unfolding of the three proteins. Only minor changes in the stability are observed. The transition midpoint (Tm) of the KH1DDKH2 and KH1KH2DD are 47 °C and 52 °C respectively, the wild type Tm KH1KH2 is 49 °C.

Figure S6: Nucleobase preference of KH1. ITC titrations of KH1KH2DD with the target RNA sequence containing single nucleotide mutations are shown. Experiments were performed as described in Methods and for each titration the baseline corrected calorimetric titration data (top) and the binding isotherm derived by area integration of peaks after each injection (bottom) is reported. The curve represents the best fit of the data using a single binding site model. K_d values are reported on each plot.

Figure S7: Chemical shift changes for UCCGU-KH1 binding. Top – Bar chart of the chemical shift changes observed upon titrating KH1KH2DD with the target UCCGU RNA sequence. The changes (Δδavg, as defined in Materials and Methods) are plotted against the protein sequence. The Δδavg of resonances that are affected by binding but whose position cannot be determined in the complex because of slow or intermediate exchange are plotted as grey bars reaching the end of the scale - as we expect to be associated with larger shifts or interface dynamics. Experiments were performed as described in the Materials and Methods section. Bottom – Residues with chemical shift changes in slow or intermediate exchange and those in fast exchange with Δδavg > 0. 15 are mapped onto the surface representation of the domain - as in Figure 3F.

Figure S8: Nucleobase preference of KH2. ITC titrations of KH1DDKH2 with the target RNA sequence containing single nucleotide mutations are shown. Experiments were performed as described in Methods and the data is presented and analysed as in Figure S5.

Figure S9: Chemical shift changes for UCCCG-KH2 binding. Top - Bar chart of the chemical shift changes observed upon titrating KH1DDKH2 with the target UCCCG RNA sequence. The changes (Δδavg, as defined in Materials and Methods) are plotted against the protein sequence. A small number Δδavg of resonances that are affected by binding but whose position cannot be determined in the complex because of intermediate exchange broadening, which we expect to be associated with larger shifts or interface dynamics, are plotted as grey bars reaching the end of the scale. Experiments were performed as described in the Materials and Methods section. Bottom – Residues with resonance chemical shift changes in intermediate or fast exchange with Δδavg > average + 1 (~0.15) are mapped onto the surface representation the domain - as in Figure 4D.

Figure S10: BLI analysis of KH1KH2 RNA binding to MYCRNA. (A) The sequence of the c-myc mRNA CRD element (chr8:128,752,990-128,753,172) is displayed, with the MYCRNA sequence highlighted in bold and underlined. The putative IMP1 KH4 binding site downstream from the MYCRNA is shown italicised and underlined. (B) BLI analysis of binding of KH1KH2DD to MYCRNA. Streptavidin-coated sensors derivatized with biotinylated MYCRNA were incubated with increasing concentrations of KH1KH2DD. Left – Raw interferograms data. Right – The fast association and dissociation phases extracted from the interferograms after the subtraction of a linear baseline slope. (C) BLI analysis of binding of wild type KH1KH2 to MYCRNA. Streptavidin-coated sensors derivatized with biotinylated MYCRNA were incubated with increasing concentrations of KH1KH2. Left – Raw interferograms data. Right - The fast association and dissociation phases extracted from the interferograms after the subtraction of the signal from the non-specific binding of free protein to the sensor and correction for a slightly sloping baseline. This non-specific signal was obtained from a protein-only control and multiplied appropriately for the different concentrations. Please note that the two highest concentrations could not be fitted using a single exponential despite the subtraction of the non-specific binding, and a double exponential fit was employed.

Supplementary figure 1

A

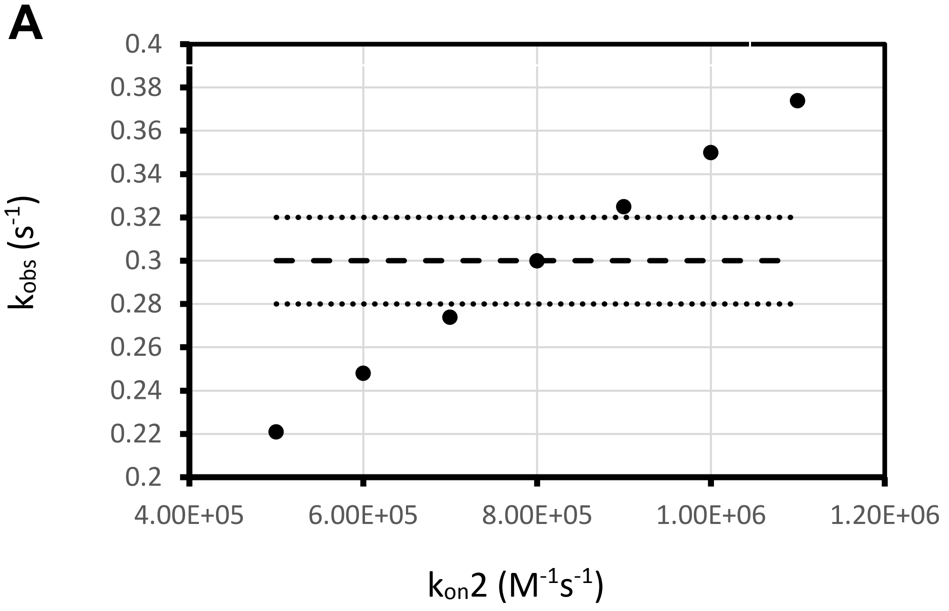

B

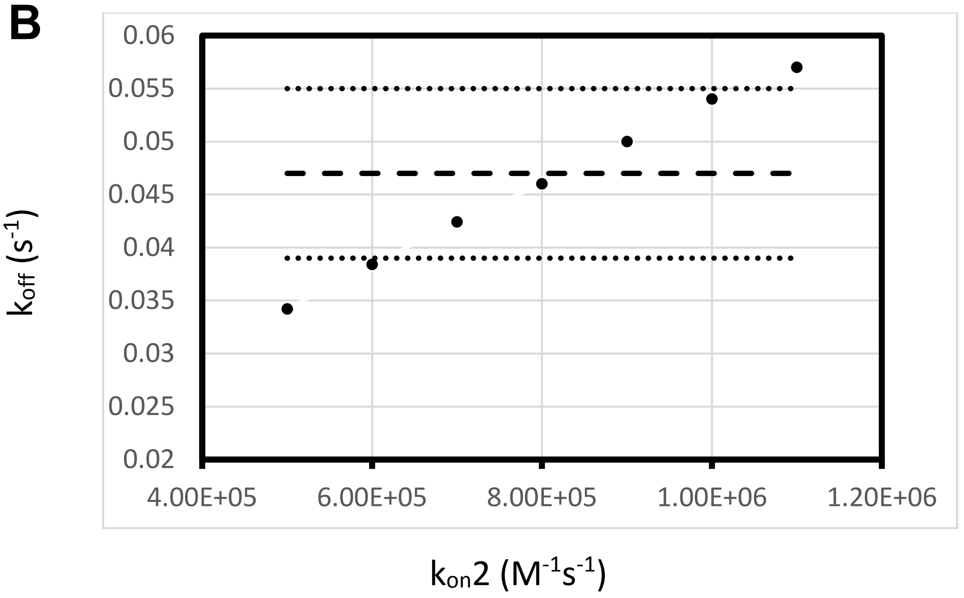

Supplementary figure 2

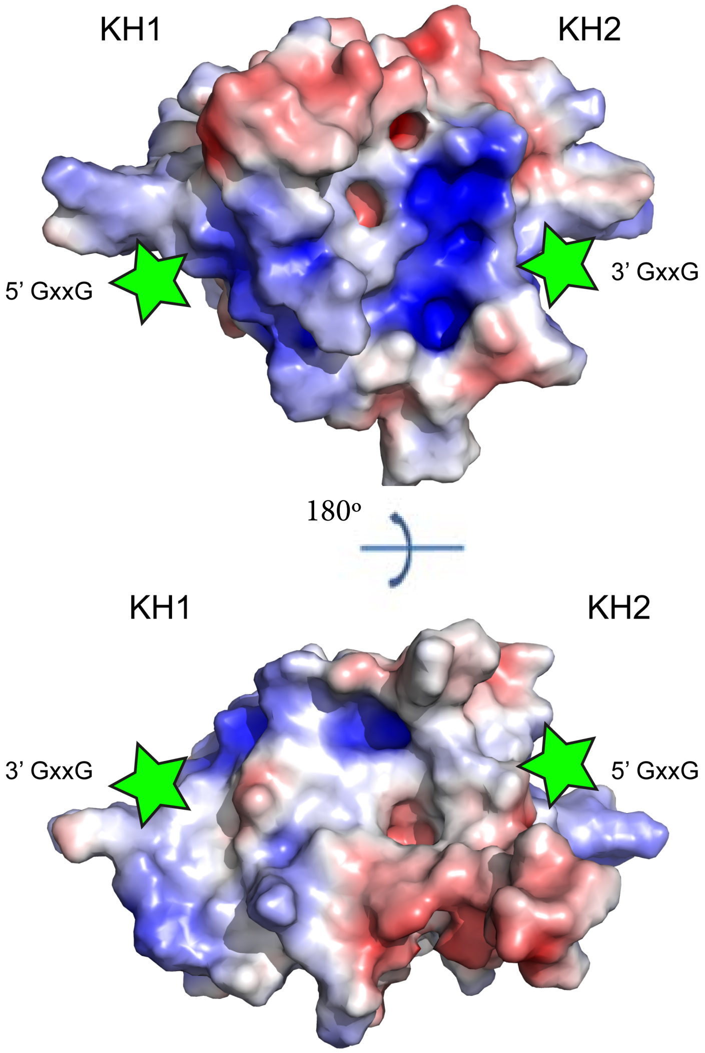

Supplementary figure 3

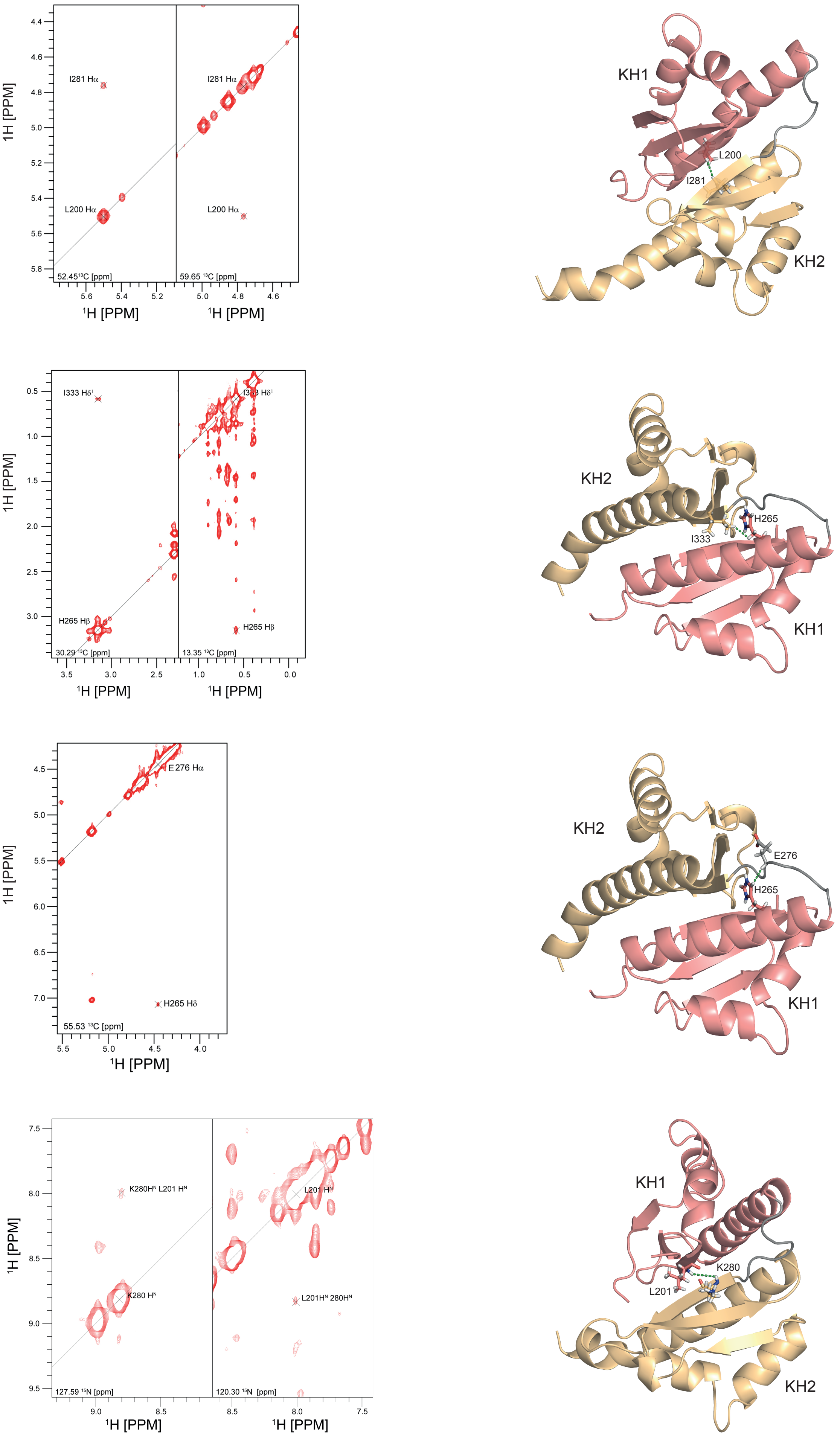

Supplementary figure 4

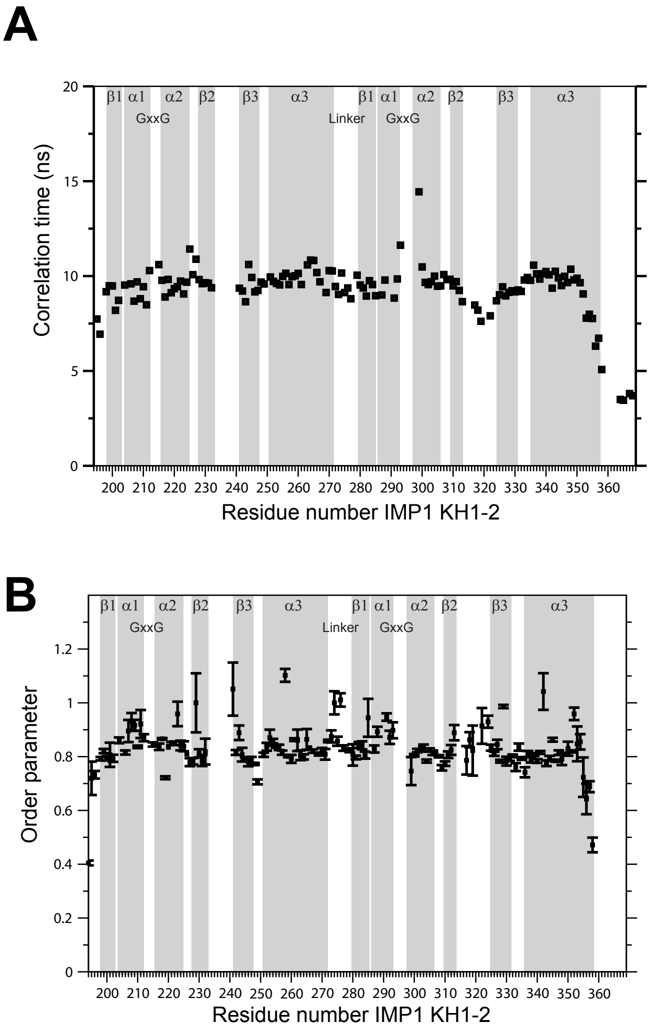

Supplementary figure 5

A

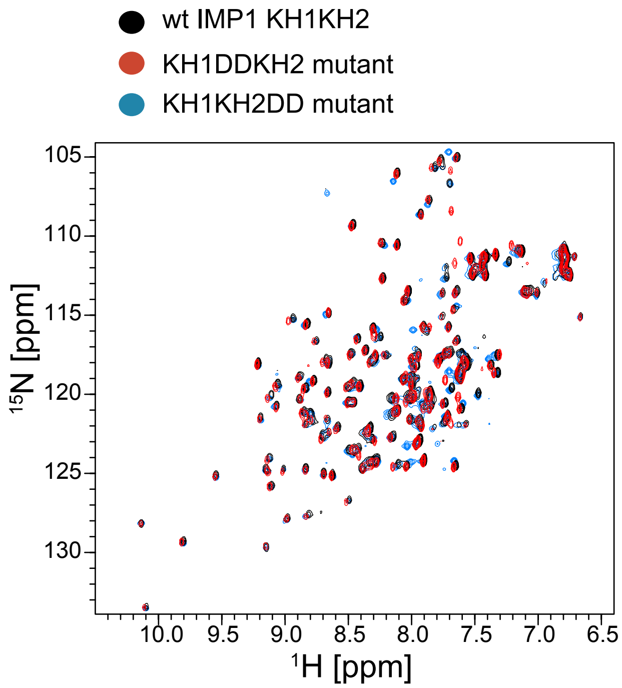

B

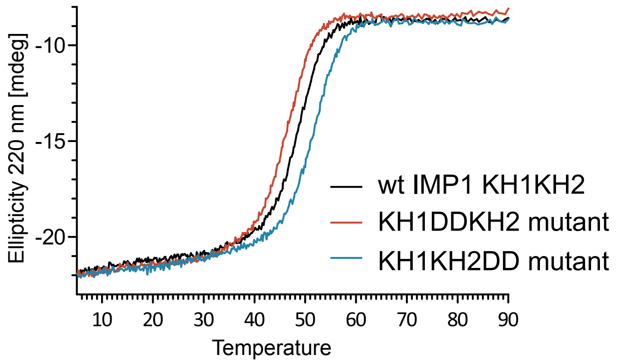

Supplementary figure 6

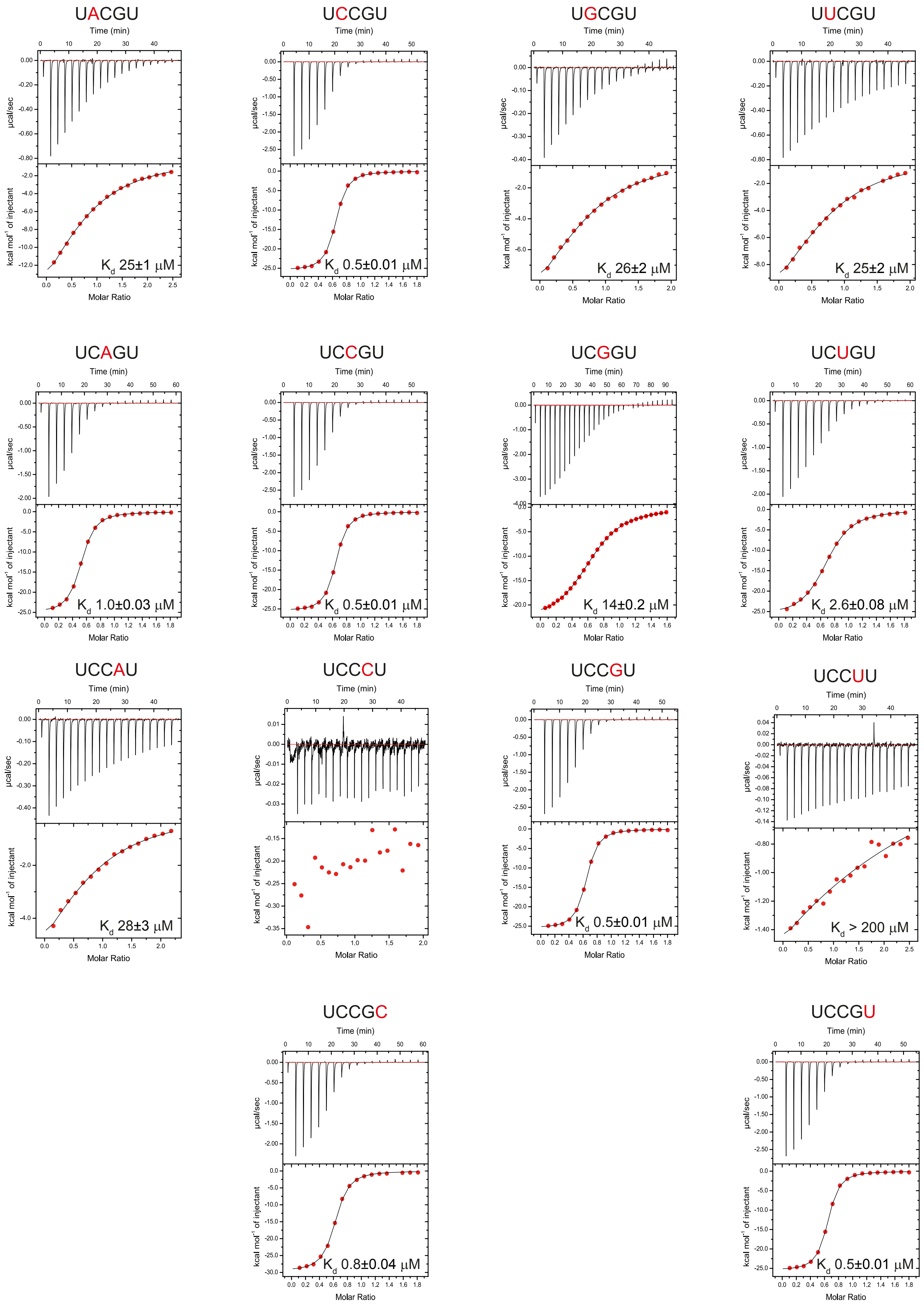

Supplementary figure 7

A

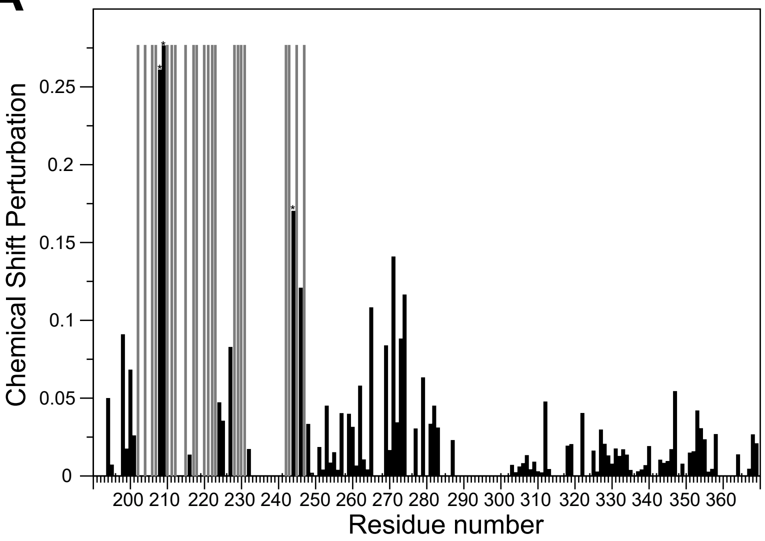

B

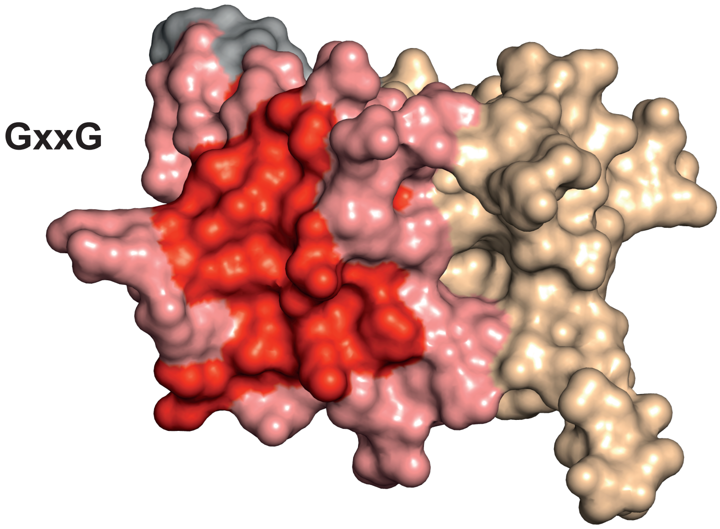

Supplementary figure 8

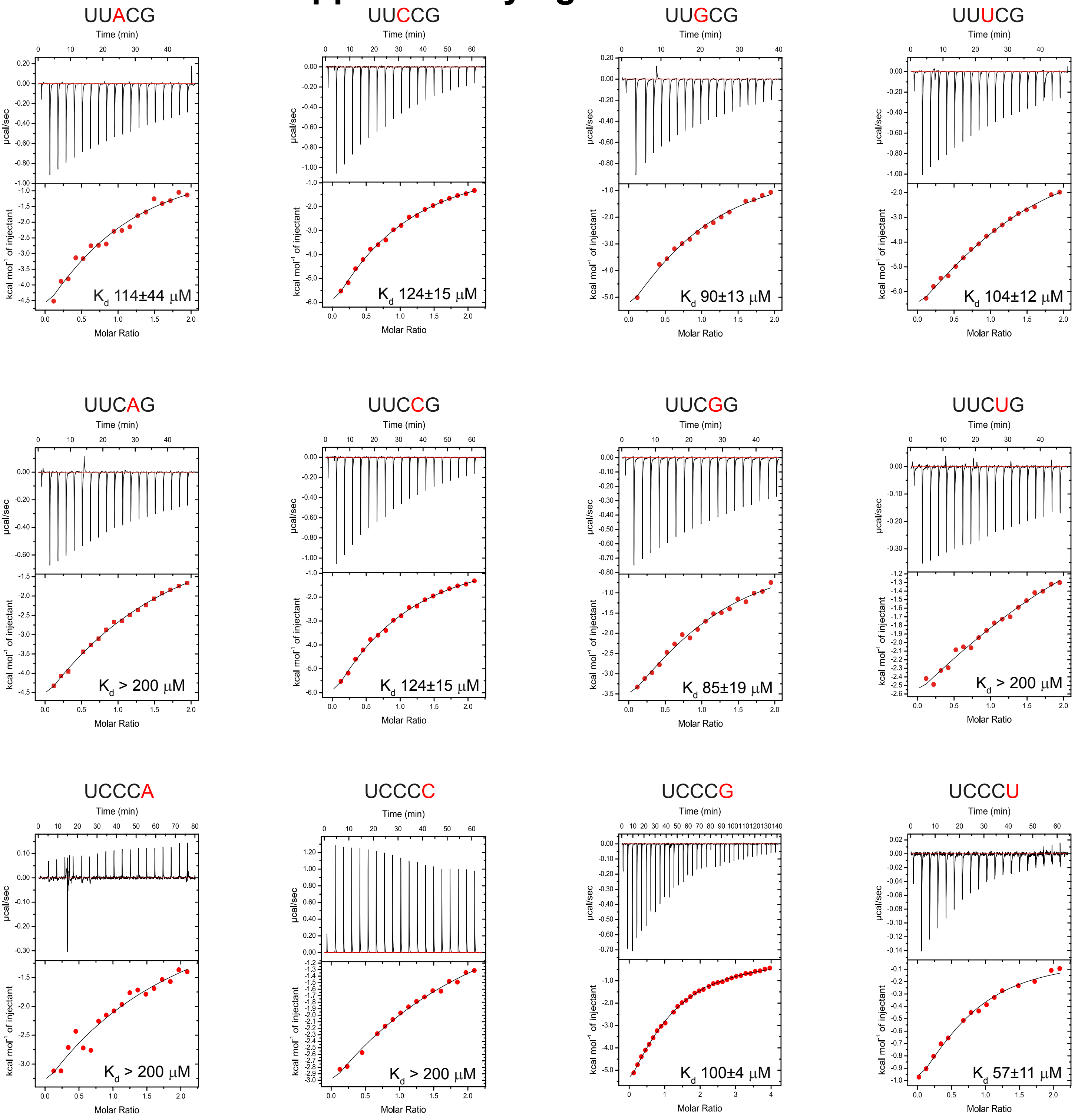

Supplementary figure 9

A

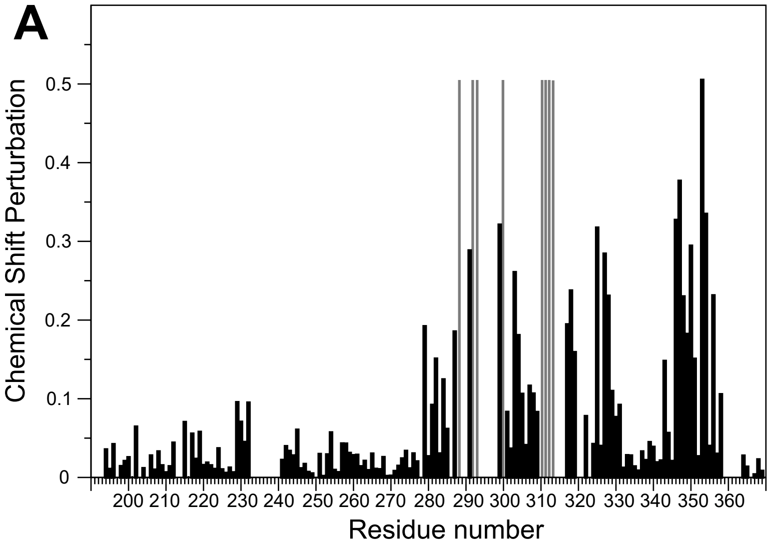

B

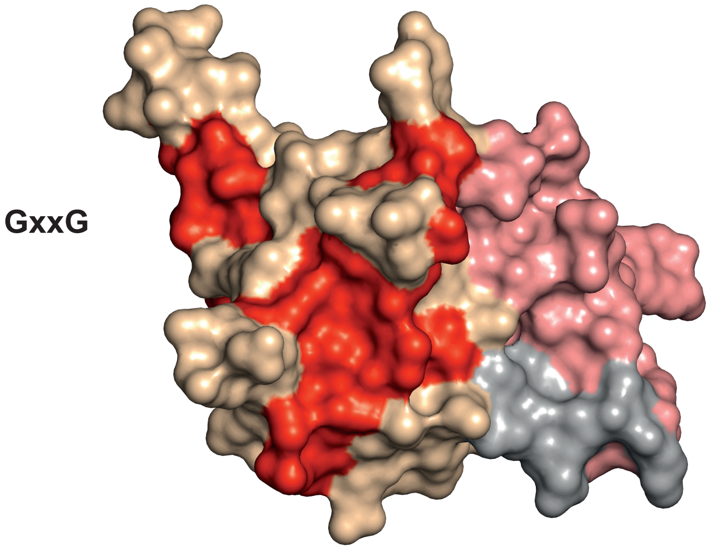

Supplementary figure 10

A

c-myc, CRD sequence

>chr8:128,752,990-128,753,172
GACCAGAUCCCGGAGUUGGAAAACAAUGAAAAGGCCCCCAAGGUAGUUAUCCUUAAAAAAG
CCACAGCAUACAUCCUGUCCGUCCAAGCAGAGGAGCAAAAGCUCAUUUCUGAAGA***GGAC***UU
GUUGCGGAAACGACGAGAACAGUUGAAACACAAA

B

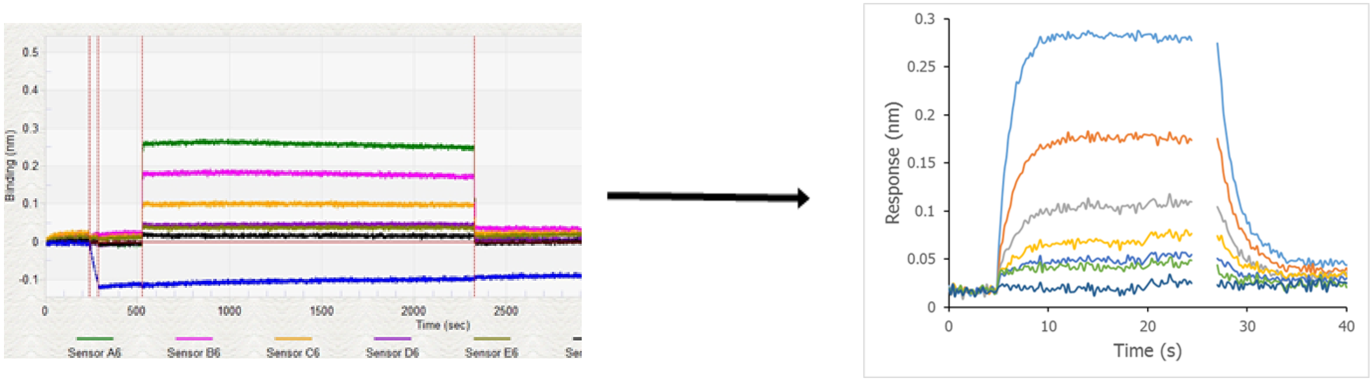

Subtraction of a constant-slope baseline and analysis of the fast association and dissociation phases.

C

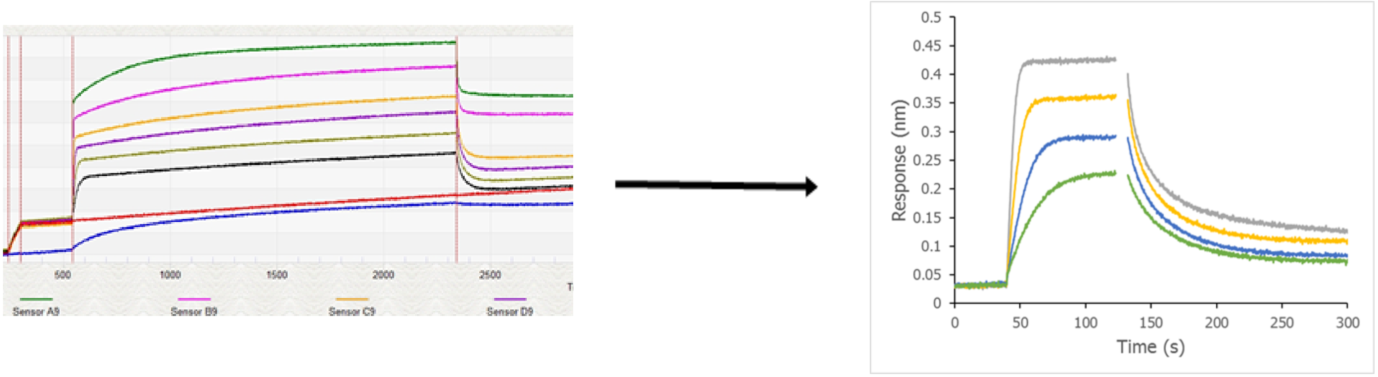

Subtraction of the non-specific binding and analysis of the fast association and dissociation phases.

Supplementary tables

Supplementary Table 1 - Values of $k_{off}2$, and kC2 obtained for different $K_d2/k_{on}2$ pairs

K_d2 M/$k_{on}2$ $M^{-1}s^{-1}$	9.1×10^4	2.7×10^5	8.2×10^5	
5.0×10^{-5}	4.6	14	41	$k_{off}2$ = kO2 (s^{-1})
	1.7×10^2	5.1×10^2	1.5×10^3	kC2 (s^{-1})
1.0×10^{-4}	9.1	27	82	$k_{off}2$ = kO2 (s^{-1})
	3.4×10^2	1.0×10^3	3.0×10^3	kC2 (s^{-1})
1.5×10^{-4}	14	41	1.2×10^2	$k_{off}2$ = kO2 (s^{-1})
	5.1×10^2	1.5×10^3	4.6×10^3	kC2 (s^{-1})

*The set of constants selected in our final model are underlined. The values obtained from the calculations have been rounded for presentation.

Supplementary Table 2 - Values of kC1 for different K_d2 values

K_d2	kC1
5.00×10^{-5} M	0.5×10^3 s^{-1}
1.00×10^{-4} M	1.1×10^3 s^{-1}
1.50×10^{-4} M	1.5×10^3 s^{-1}

* The Kd2 value selected in our final model is underlined. The values obtained from the calculations have been rounded for presentation.

Supplementary Table 3 - % Saturation of RNA at different values of K_d

K_d	% saturation RNA
20 nM	90.9%
40 nM	83.3%
80 nM	73.3%

*Protein concentration = 200 nM
